# Older adults at greater risk for Alzheimer’s disease show stronger associations between sleep apnea severity in REM sleep and verbal memory

**DOI:** 10.1186/s13195-024-01446-3

**Published:** 2024-05-09

**Authors:** Kitty K. Lui, Abhishek Dave, Kate E. Sprecher, Miranda G. Chappel-Farley, Brady A. Riedner, Margo B. Heston, Chase E. Taylor, Cynthia M. Carlsson, Ozioma C. Okonkwo, Sanjay Asthana, Sterling C. Johnson, Barbara B. Bendlin, Bryce A. Mander, Ruth M. Benca

**Affiliations:** 1 San Diego State University/University of California San Diego, Joint Doctoral Program in Clinical Psychology, San Diego, CA USA; 2 Department of Psychiatry and Human Behavior, University of California, Irvine, CA USA; 3 Department of Cognitive Sciences, University of California, Irvine, CA USA; 4 Department of Population Health Sciences, University of Wisconsin-Madison, Madison, WI USA; 5 Neuroscience Training Program, University of Wisconsin-Madison, Madison, WI USA; 6 Department of Medicine, University of Wisconsin-Madison, Madison, WI USA; 7 Wisconsin Alzheimer’s Disease Research Center, University of Wisconsin-Madison, Madison, WI USA; 8 Department of Neurobiology and Behavior, University of California, Irvine, CA USA; 9 Center for the Neurobiology of Learning and Memory, University of California, Irvine, CA USA; 10 Department of Psychiatry, University of Wisconsin-Madison, Madison, WI USA; 11 Department of Neuroscience, University of Kentucky, Lexington, KY USA; 12 Wisconsin Alzheimer’s Institute, Madison, WI USA; 13 Geriatric Research Education and Clinical Center, Wm. S. Middleton Veterans Hospital, Madison, WI USA; 14 Department of Psychiatry and Behavioral Medicine, Wake Forest University, Winston-Salem, NC USA

## Abstract

**Background:**

Obstructive sleep apnea (OSA) increases risk for cognitive decline and Alzheimer’s disease (AD). While the underlying mechanisms remain unclear, hypoxemia during OSA has been implicated in cognitive impairment. OSA during rapid eye movement (REM) sleep is usually more severe than in non-rapid eye movement (NREM) sleep, but the relative effect of oxyhemoglobin desaturation during REM versus NREM sleep on memory is not completely characterized. Here, we examined the impact of OSA, as well as the moderating effects of AD risk factors, on verbal memory in a sample of middle-aged and older adults with heightened AD risk.

**Methods:**

Eighty-one adults (mean age:61.7 ± 6.0 years, 62% females, 32% *a*polipoprotein E ε4 allele (*APOE4)* carriers, and 70% with parental history of AD) underwent clinical polysomnography including assessment of OSA. OSA features were derived in total, NREM, and REM sleep. REM-NREM ratios of OSA features were also calculated. Verbal memory was assessed with the Rey Auditory Verbal Learning Test (RAVLT). Multiple regression models evaluated the relationships between OSA features and RAVLT scores while adjusting for sex, age, time between assessments, education years, body mass index (BMI), and *APOE4* status or parental history of AD. The significant main effects of OSA features on RAVLT performance and the moderating effects of AD risk factors (i.e., sex, age, *APOE4* status, and parental history of AD) were examined.

**Results:**

Apnea–hypopnea index (AHI), respiratory disturbance index (RDI), and oxyhemoglobin desaturation index (ODI) during REM sleep were negatively associated with RAVLT total learning and long-delay recall. Further, greater REM-NREM ratios of AHI, RDI, and ODI (i.e., more events in REM than NREM) were related to worse total learning and recall. We found specifically that the negative association between REM ODI and total learning was driven by adults 60 + years old. In addition, the negative relationships between REM-NREM ODI ratio and total learning, and REM-NREM RDI ratio and long-delay recall were driven by *APOE4* carriers.

**Conclusion:**

Greater OSA severity, particularly during REM sleep, negatively affects verbal memory, especially for people with greater AD risk. These findings underscore the potential importance of proactive screening and treatment of REM OSA even if overall AHI appears low.

**Supplementary Information:**

The online version contains supplementary material available at 10.1186/s13195-024-01446-3.

## Background

Obstructive sleep apnea (OSA) is characterized by recurrent pharyngeal airway collapses that cause complete (apneas) or partial (hypopneas) cessations of airflow that lead to sleep fragmentation and intermittent hypoxemia [1]. Meta-analyses have shown that sleep-disordered breathing (SDB), including OSA, increases incidence of Alzheimer’s disease (AD), and people with AD were five times more likely to have OSA [2, 3]. Proposed mechanisms suggest that OSA accelerates expression of AD pathologies, medial temporal lobe (MTL) degeneration, and memory impairment through OSA-related hypoxemia [4–8]. Though, some have reported that the cognitive consequences of OSA are diminished in older age [9]. Verbal memory deficits, specifically learning and recall of word list, is considered as the most sensitive marker of early cognitive changes associated with AD [10–12], and OSA-related hypoxemia could exacerbate AD risk through its impact on the hippocampus, a brain region critical for the formation and processing of episodic memories and is especially vulnerable to injury from oxygen deprivation [13, 14]. While OSA severity has been linked to poor verbal memory performance, especially word lists learning tests [8, 15], the exact OSA features driving these relationships, as well as whether respiratory events occurring during non-rapid eye movement (NREM) or rapid eye movement (REM) sleep are more damaging, have remained unclear.

During REM sleep, there is higher neurometabolic demand in regions impacted in early AD compared to NREM sleep [16–18]. In addition, during REM sleep, there is an increased susceptibility of upper airway collapse due to inhibition of the genioglossus muscle (the major upper airway dilator muscle that helps stabilize breathing) [19]. There are also lower hypoxic and hypercapnic respiratory drives during REM sleep which results in longer durations of apneas and hypopneas, and more instances of oxyhemoglobin desaturation than in NREM sleep [20]. These specific features of REM sleep and OSA events during REM sleep allude to the possibility that OSA during REM sleep may impart greater cognitive consequences, however, this has yet to be fully examined.

Furthermore, AD risk factors, such as older age, female sex, and apolipoprotein E ε4 (*APOE4*) genotype have been implicated in OSA as well. OSA and AD are both more prevalent in the aging population [21]. Roughly 40–80% of people with AD carry at least one *APOE4* allele and older adults with *APOE4* may have increased risk for SDB, although this has not been consistently reported [22, 23]. Moreover, the effects of *APOE4* on the associations between OSA and memory have remained unclear [24, 25], though it appears that OSA’s effects on memory networks may be stronger in β-amyloid positive older adults [26]. There are also sex differences in both AD and OSA risk. Women are nearly twice as likely to be diagnosed with AD and have a more severe disease progression that is characterized by faster memory decline and more pathological tau accumulation [27–30]. While men are at increased risk for OSA, OSA prevalence substantially rises in post-menopausal women, yet women typically remain underdiagnosed [31, 32]. There is some evidence that suggests women with OSA may develop stronger OSA-related memory impairments than men with OSA, though, results have been inconsistent [33–35]. OSA is also expressed differentially by sex, with apneas and hypopneas more likely to occur in REM sleep in women than men [36, 37]. Thus, there is a possibility of interplay between OSA and AD risk factors of age, sex, and *APOE4*, that may synergistically cause stronger memory impairments. However, these interactions have yet to be fully examined.

Here, in the current study, we sought to examine OSA expression separately during REM and NREM sleep as it related to verbal memory, and whether AD risk factors moderated these relationships. We combined clinical polysomnography (PSG) with verbal memory measured by the Rey Auditory Verbal Learning Test (RAVLT) in a cohort of middle- and older- aged adults enriched for parental history and genetic risk for AD. We aimed to extend the current literature by testing the following hypotheses: 1) greater OSA severity, particularly during REM sleep, is associated with impaired verbal learning and delayed recall and 2) that in significant associations, the relationships are stronger among older adults, women, and/or individuals with increased genetic and parental risk for AD.

## Methods

### Clinical methods

One hundred fifteen cognitively unimpaired middle- and older-aged adults from the Wisconsin Alzheimer’s Disease Research Center (ADRC) Clinical Core, a prospective cohort study enriched for probable parental history of AD relative to the general population [38], were enrolled in a sub-study, the Predicting Alzheimer’s from Metabolic Markers and Sleep (PAMMS). From their ADRC visit, participants underwent cognitive assessments of declarative and semantic memory, attention, executive function, language, and visuospatial processing using the National Alzheimer’s Coordinating Center Uniform Data Set (UDS) neuropsychological battery version 3 and additional assessments [39]. Clinical diagnosis of cognitively unimpaired status was determined using the 2011 National Institute on Aging-Alzheimer’s Association (NIA-AA) workgroup diagnostic criteria and confirmed by multidisciplinary consensus conference (Table S1) [40, 41].

Of the 115 participants in PAMMS, 89 underwent a subcomponent of the study with polysomnography (PSG) with high-density EEG (hdEEG). This portion of the study excluded for individuals with a past history or current neurological, psychiatric, medical conditions, or treatments that impacted their cognition, or hindered their ability to complete any aspects of the study protocol, taking medications known to influence sleep or sleep electroencephalography (EEG), including antipsychotic medications, non-selective serotonin reuptake inhibitors (SSRIs) antidepressants, neuroleptics, chronic anxiolytics, sedative hypnotics, and stimulants, and was currently undergoing treatment for SDB (e.g. continuous positive airway pressure). Participants completed PSG with hdEEG approximately within 1 year of cognitive assessment. RAVLT scores were taken from their cognitive assessment at the ADRC. In addition, participant’s *APOE*4 genotyped by DNA extraction from whole blood samples using competitive allele-specific PCR based KASP genotyping for rs429358, as previously reported [42]. The final sample size consisted of 81 participants with valid PSG data and RAVLT scores (e.g., oxyhemoglobin saturation levels not recorded the whole night would be considered invalid data).

### Polysomnography

To assess sleep and OSA severity, participants underwent clinical PSG with 256-channel hdEEG. A thorough description of PSG with hdEEG recording and sleep scoring has been previously described [43]. From PSG, sleep architecture measures of total sleep time (TST), time in bed (TIB), sleep onset latency, wake after sleep onset (WASO), and percent of TST spent in N1, N2, N3, and REM were derived. Additionally, clinical measures reflecting sleep disorder characteristics were calculated, including apnea–hypopnea index (AHI; number of apneas and hypopneas per hour), respiratory disturbance index (RDI; number of apneas, hypopneas, and respiratory-related arousals per hour), oxyhemoglobin desaturation index (ODI; number of oxyhemoglobin desaturations ≥ 4% per hour). The AHI is a traditional measure for OSA diagnosis, with the American Academy of Sleep Medicine (AASM) clinical criterion of an AHI ≥ 5 for at least mild OSA [44, 45]. The RDI captures additional information regarding respiratory events that do not meet criteria for a hypopnea, yet still lead to an arousal, and thus disrupt continuous sleep [44, 45]. The AASM criteria for OSA diagnosis is RDI ≥ 5, if daytime sleepiness is present, and RDI ≥ 15 if not. The ODI is a clinically informative measure on the frequency of drops in oxyhemoglobin saturation levels, a marker of intermittent hypoxemia, which has been associated with poor cardiovascular outcomes, including elevated stroke risk, and increased risk of mortality [46–48].

Furthermore, nadir blood oxyhemoglobin saturation level, mean blood oxyhemoglobin saturation, duration of time spent with < 90% blood oxyhemoglobin saturation, and periodic leg movements during sleep index (PLMSI; number of PLMs per hour) were analyzed. These measures besides PLMSI, also reflects the degree of hypoxemia that occurs during sleep, and are additionally diagnostically important for OSA [45, 47]. This study included participants with and without OSA and used the clinical measures of OSA as continuous variables for statistical analyses.

In addition, AHI, RDI, ODI, and duration of time spent with < 90% oxyhemoglobin saturation in REM and NREM sleep were also derived. To measure whether an individual had more REM or NREM OSA features throughout the night, ratios of AHI, RDI, and ODI between REM and NREM sleep were also calculated [49–52]. The NREM/REM ratios provide context of whether there is predominance of OSA events in a specific sleep stage across the sleep period. There are well characterized physiological differences in REM versus NREM sleep; specifically, OSA in REM sleep leads to more severe OSA events and has been linked to poor cardiovascular outcomes [7, 16–20, 53–56]. While some studies reported no significant differences between those with OSA events predominantly in NREM sleep versus REM sleep in clinical features such as BMI, daytime sleepiness, and depression [50, 51], there have been established physiological and polysomnographic differences in those that express OSA events more in REM sleep and in those that express OSA events more in NREM sleep [51, 52]. For instance, in predominant NREM OSA, ventilatory control is worse with higher loop gain, whereas in predominant REM OSA, the upper airway is more collapsible. Furthermore, people with more predominant NREM OSA had longer sleep onset latency, less sleep efficiency, and lower mean oxyhemoglobin saturation. There has yet to be an examination of the ratio of REM OSA events to NREM OSA event as it relates to memory.

### Rey Auditory Verbal Learning Test (RAVLT)

The RAVLT is a standard neuropsychological assessment for verbal memory that it MTL-dependent, a sensitive marker of memory impairment in preclinical AD, and commonly used in AD research and clinical practice for diagnosis [57–60]. Further, OSA severity has been linked to poor verbal memory performance [8].

The test includes one learning phase, two recall phases, and one recognition phase. During the learning phase, a list of 15 words is read to the participant five times, and the participant repeat the words they remember after each trial. An interference list of 15 words is then read aloud once, and the short-delay recall ability is assessed after the interference list. Long-delay recall is then assessed after 20 min. A total learning score is derived by summing the number of remembered words in trials 1 through 5 (range: 0–75). Short-delay recall was measured as total number of words recalled after the interference list (range: 0–15), and long-delay recall was measured as total number of words after the 20-min delay (range: 0–15).

### Statistical analyses

The purpose of this study was to comprehensively examine the distinct features of OSA (e.g., number of OSA-related events per hour or time spent during sleep with blood oxyhemoglobin levels in hypoxemia) across the whole night and broken down by distinct sleep stages (NREM versus REM), since there are physiological differences in these brain states [16–20]. Thus, the OSA characteristics that were analyzed were AHI, RDI, and ODI, and sleep duration spent with < 90% blood oxyhemoglobin saturation for total sleep, REM sleep, and NREM sleep. Total sleep nadir blood oxyhemoglobin saturation, total sleep mean blood oxyhemoglobin saturation, and WASO were also analyzed. RAVLT measures included total learning, short-delay recall, and long-delay recall. Normality of variables were analyzed with a Shapiro–Wilk test. All AHI, RDI, and ODI measures were log-transformed with a constant added to meet normality assumptions.

Independent sample t-tests were conducted to analyze group differences by sex, *APOE4* status, and parental history of AD. Student’s t-tests were used if assumptions of normality and variance were met. Mann Whitney U-tests were used if assumptions of normality were violated. Kendall rank correlation was conducted on the associations between age and OSA characteristics. Paired samples t-tests were used to analyze REM versus NREM AHI, RDI, ODI, and duration spent with < 90% blood oxyhemoglobin saturation. Student’s t-test was used if assumptions of normality and Wilcoxon signed-rank test was used if assumptions of normality were violated.

Multiple linear regression models were used to analyze the relationships between OSA characteristics (predictors) and RAVLT (outcomes), controlling for sex, age, time between PSG and RAVLT, *APOE4* status, body mass index (BMI), and years of education. One participant’s BMI was not measured and another's *APOE4* status was not obtained, and thus were not included in statistical analyses that included those measures. Across all 15 models, the Benjamin-Hochberg method for False Discovery Rate (FDR) correction was used to correct multiple comparisons [61]. Regressions were repeated, substituting *APOE4* status for parental history of AD.

To further understand the relative impact of OSA during REM sleep against NREM sleep on verbal memory performance, post hoc analyses included using the Steiger’s Z test to directly compare the correlation strengths of the associations between REM and NREM sleep apnea features and RAVLT scores [62]. Also, REM-NREM AHI, RDI, and ODI ratios were calculated, and the ratios were log-transformed with a constant added to meet normality assumptions. Multiple linear regression models were then used to analyze the associations between the OSA feature by sleep stage ratios and RAVLT scores while controlling for the same covariates. Regressions were repeated substituting *APOE4* status for parental history of AD as a covariate.

For significant sleepstage findings, we wanted to account for the possible effects of sleep duration in that sleep stage on the significant associations between OSA features and RAVLT measures. Thus, we conducted follow-up analyses that included percent of time spent in that sleep stage in the models. Also, to disentangle whether total number of OSA events in that sleep stage or sleep duration in that sleep stage (i.e., the values that go into calculating AHI/RDI/ODI) was driving the detected effects, we conducted linear regression models with those separate measures as predictors. Total number of apnea/hypopneas, respiratory disturbances, and oxyhemoglobin desaturations during REM sleep were all log-transformed to meet assumptions of normality.

For significant associations between OSA characteristics and verbal memory, we investigated the moderating effects of sex, age, *APOE4* status, and parental history of AD (*APOE4* status and parental history of AD in separate regression models) on those relationships. To further probe significant interactions with either *APOE4* status or parental history of AD, we grouped participants into three groups consisting of 1) people with no *APOE4* or parental history of AD, 2) people with either *APOE*4 or parental history of AD, and 3) people with both *APOE4* and parental history of AD. Analysis of covariance (ANCOVA) was used to analyze interactions between AD risk group and OSA features as it predicted verbal memory while controlling for the same covariates mentioned above. Slopes of the relationship between OSA features and verbal memory between the 3 groups were compared and Tukey’s method was used to correct for multiple comparison [63]. For significant interactions with age, Johnson-Neyman intervals and simple slope analyses were used to determine how much of the sample was driving the significant moderating effect [64–66]. All statistical analysis was conducted on JASP (Version 0.17.3) and RStudio (Version 2021.09.2).

## Results

### Sample characteristics

Participant demographics and RAVLT scores of the 81 participants are shown in Table 1. Sleep architecture and OSA characteristics are shown in Table 2. In this sample, the average age was 61.7 ± 6.0 years (age range: 44–88 years), 60% participants were female, 32.5% of them were *APOE4* carriers, 69.1% had parental history of AD, and 26.3% were both *APOE4* carriers and had parental history of AD. The average time between PSG and RAVLT assessments was 0.31 ± 0.50 years. Nearly half of the cohort (44.44%) had OSA (AHI ≥ 5/h) and 16.05% had moderate or severe OSA (AHI ≥ 15/h). OSA severity was significantly higher in REM sleep than in NREM sleep (AHI: (t(80) = 7.91, *p < *0.001), RDI: (t(81) = 5.11, *p < *0.001), ODI: (t(81) = 9.64, *p < *0.001). Remarkably, the duration spent with blood oxyhemoglobin levels < 90% did not differ significantly between REM and NREM sleep stages (1.83 ± 4.57 versus 2.55 ± 7.21, z = 0.06, *p = *0.96), despite the fact that participants spent significantly more of the sleep period in NREM than in REM sleep stages (NREM:279.49 ± 57.71 min versus REM:59.80 ± 28.69 min, t(80) = 36.17, *p < *0.001). This resulted in the proportion of time spent with blood oxyhemoglobin levels < 90% being significantly higher during REM than in NREM sleep stages (REM: 0.04 ± 0.11 versus NREM:0.01 ± 0.03, z = 3.68, *p < *0.001).

**Table 1:** Participant descriptive characteristics (*n = *81)

	**Mean [SD]**
**Demographics**	
PSG age (years)	61.68 [6.0]
RAVLT age (years)	61.38 [6.13]
Time between PSG and RAVLT	0.31 [0.50]
Female (n; %)	49; 60%
*APOE* ε4-positive genotype (n; %)	26; 32%
Parental history of AD (n; %)	56; 69%
Both *APOE* ε4-positive genotype and parental history of AD	21; 26%
Years of education	16.43 [2.41]
BMI	28.10 [5.53]
**Primary Race**	
White (n; %)	71; 87.8%
Black or African American (n; %)	9; 11.1%
Asian (n; %)	1; 1.2%
**Ethnicity**	
Non-Hispanic (n; %)	79; 97.5%
Hispanic (n; %)	1; 1.2%
Unknown (n; %)	1; 1.2%
**RAVLT**	
Total Learning	51.63 [10.48]
Short-Delay Recall	10.77 [2.89]
Long-Delay Recall	10.22 [3.17]

*PSG* polysomnography, *RAVLT* Rey Auditory Verbal Learning Test, *APOE* apolipoprotein E, *AD* Alzheimer’s Disease, *APOE4* genotyping missing from one participant, *BMI* missing from one participant

**Table 2:** Participant sleep architecture and OSA characteristics (*n = *81)

	**Mean [SD]**
**Sleep architecture**	
Total time in bed (minutes)	451.81 [65.50]
Total sleep time (minutes)	339.30 [72.92]
Sleep onset latency (minutes)	22.98 [24.04]
Sleep efficiency (%)	75.64 [14.35]
Wake after sleep onset (minutes)	89.54 [59.70]
Stage NREM 1 (%)	9.59 [9.91]
Stage NREM 2 (%)	59.99 [10.80]
Stage NREM 3 (%)	13.26 [10.31]
Stage REM (%)	17.03 [6.32]
Periodic Limb Movement Index	15.33 [19.37]
**OSA characteristics**	
Apnea–Hypopnea Index (AHI)	8.26 [12.34]
REM AHI	17.19 [20.99]
NREM AHI	6.42 [12.15]
REM AHI:NREM AHI	4.02 [4.55]
Respiratory Disturbance Index (RDI)	15.96 [16.58]
REM RDI	24.19 [22.58]
NREM RDI	14.20 [16.65]
REM RDI:NREM RDI	2.53 [2.40]
Oxyhemoglobin Desaturation Index (ODI)	12.69 [15.30]
REM ODI	23.80 [21.10]
NREM ODI	10.41 [15.39]
REM ODI:NREM ODI	3.60 [2.97]
Nadir Blood Oxyhemoglobin saturation (%)	85.62 [7.98]
Mean Blood Oxyhemoglobin saturation (%)	94.78 [1.69]
Duration of < 90% Blood Oxyhemoglobin saturation (minutes)	4.37 [9.24]
REM < 90% Blood Oxyhemoglobin saturation (minutes)	1.83 [4.57]
NREM < 90% Blood Oxyhemoglobin saturation (minutes)	2.55 [7.21]

*REM* rapid eye movement, *NREM* non-rapid eye movement, *AHI* apnea-hypopnea index, *RDI* respiratory disturbance index, *ODI* oxyhemoglobin desaturation index

### Sex, age, APOE4 status, parental history of AD effects on OSA

Overall, male participants had more severe OSA than females (see Table S2). However, females did have a significantly higher REM-NREM ODI ratio (t(79) = 2.45, *p = *0.02). There were no significant associations observed between age and any OSA characteristics (all *p > *0.10; see Table S3). *APOE4* carriers had significantly lower overall AHI, RDI, and ODI; REM AHI, as well as lower NREM AHI and ODI when compared to *APOE4* non-carriers (all *p < *0.05; see Table S4), indicating that in this cohort, *APOE4* carriers did not have greater OSA severity compared to non-carriers. There was also no significant difference in OSA characteristics among participants with and without a parental history of AD (all *p > *0.30; see Table S5). These findings indicate that those with higher AD risk did not show evidence of greater OSA severity in this cognitively intact cohort.

### Sex, age, APOE4 status, parental history of AD effects on verbal memory

As previously reported, females had higher RAVLT scores than males (all *p < *0.05; see Table S6) [67]. Age and verbal memory were not significantly correlated (all *p > *0.10; see Table S7) and RAVLT scores did not significantly differ between *APOE4* carriers and non-carriers (all *p > *0.20; see Table S8). Interestingly, participants with parental history of AD performed better across the RAVLT compared to those without parental history of AD (all *p < *0.002; see Table S9).

### Associations between sleep apnea and verbal memory and total learning

**Fig. 1:**
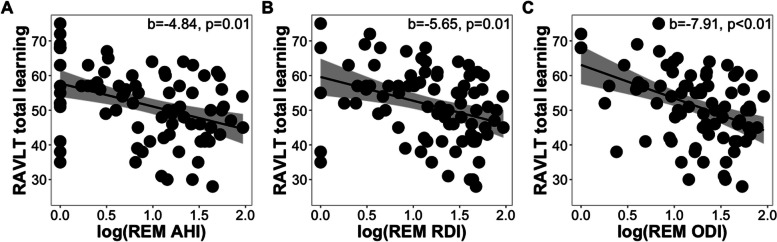
Scatter plots showing the relationships of (**A**) AHI, **(B**) RDI, and (**C**) ODI during REM sleep to RAVLT total learning scores while controlling for age, sex, time between assessments, years of education, BMI, and *APOE4* status

Total AHI (b = -4.47, *p = *0.09), RDI (b = -3.70, *p = *0.17, and ODI (b = -5.17, *p = *0.09) were not significantly associated with total learning. Similar results were observed when substituting *APOE4* status with parental history of AD in the models. When testing OSA metrics by sleep stage, we found that REM AHI (b = -4.84, *p = *0.01, FDR-corrected *p = *0.07), REM RDI (b = -5.65, *p = *0.01, FDR-corrected *p = *0.06), and REM ODI (b = -7.91, *p = *0.001, FDR-corrected *p = *0.02) were all significantly associated with total learning, with REM ODI as the only significant predictor following FDR correction (Fig. 1A-C). In the models with parental history of AD as the covariate instead of *APOE4* status, REM RDI and REM ODI were significant predictors of total learning after FDR correction (all FDR-corrected *p < *0.05). However, these same features in NREM sleep were not significantly associated with total learning performance after adjusting for covariates (NREM AHI: (b = -1.32, *p = *0.63), NREM RDI: (b = -1.47, *p = *0.56), NREM ODI: (b = -2.41, *p = *0.40)). In addition, WASO, TST nadir and average oxyhemoglobin desaturation, and duration spent with < 90% blood oxyhemoglobin saturation in TST, NREM, and REM were not significantly associated with total learning (all *p > *0.40; See Table S10 for full statistical details). Similar insignificant results were found with models that included parental history of AD instead of *APOE4* status (all ps > 0.30; See Table S11 for full statistical details).

**Fig. 2:**
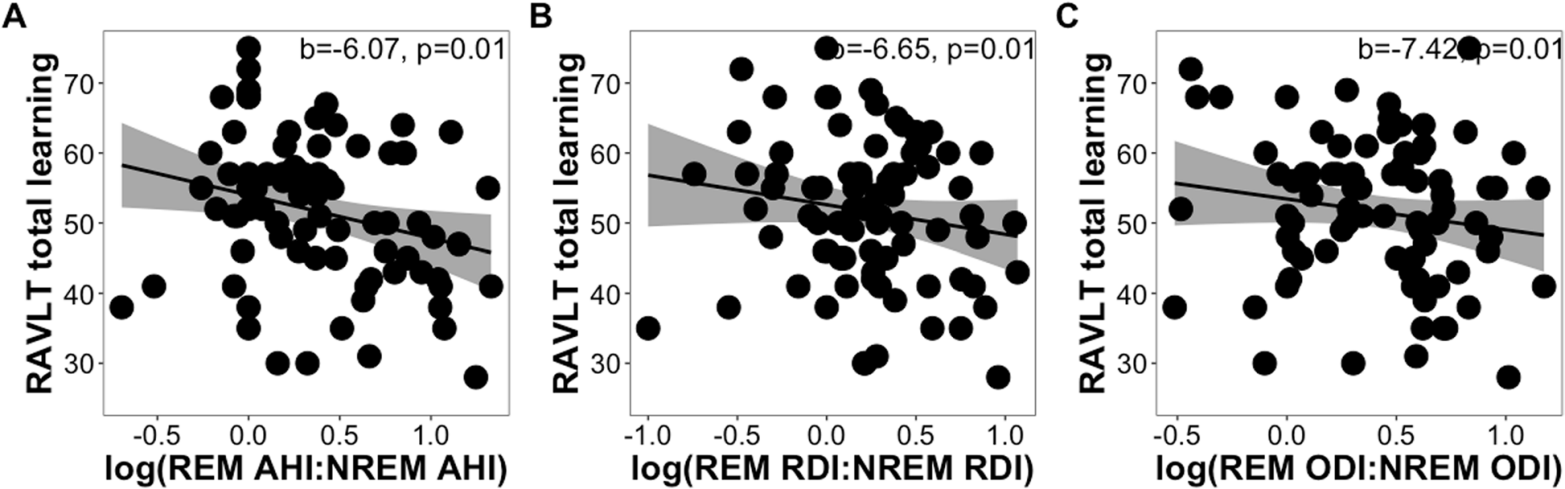
Scatter plots showing the relationships of REM-NREM (**A**) AHI, (**B**) RDI, and (**C**) ODI ratios to RAVLT total learning scores while controlling for age, sex, time between assessments, years of education, BMI, and *APOE4* status

Steiger’s Z test revealed that correlations between total learning and AHI (z = 1.66, *p = *0.10), RDI (z = 1.29, *p = *0.20), and ODI (z = 1.51, *p = *0.13) did not differ significantly between REM and NREM sleep. Although there were no significant differences in correlation strengths between REM and NREM OSA features in their associations with RAVLT total learning, the regression models indicated that REM OSA features, especially REM ODI, were significant predictors of verbal learning deficits, whereas NREM OSA features were not. Further, multiple regression models revealed that the ratios of REM-NREM AHI (b = -6.07, *p = *0.01), RDI (b = -6.65, *p = *0.01), and ODI (b = -7.42, *p = *0.01) were significantly associated with total learning (Fig. 2A-C; see Table S12). Consistent results were found in models with parental history of AD instead of *APOE4* status as the covariate (all *p < *0.02; see Table S13). These findings indicated that greater OSA severity during REM sleep in comparison to NREM sleep was associated with diminished total learning performance.

### Associations between sleep apnea and short-delay recall

Overall AHI (b = -0.84, *p = *0.27), RDI (b = -0.44, *p = *0.56), and ODI (b = -0.80, *p = *0.37) were not significantly related to short-delay recall. REM AHI (b = -1.38, *p = *0.01, FDR-corrected *p = *0.21) and REM ODI (b = -1.54, *p = *0.03, FDR-corrected *p = *0.23) were associated with short-delay recall but were no longer significant after FDR correction (See Table S14 for full statistical details of all predictors). In models with parental history of AD instead of *APOE4* status, REM AHI was associated with short-delay recall (*p = *0.04; FDR-corrected *p = *0.48), but not after FDR correction (See Table S15 for full statistical details of all predictors). Steiger’s Z test revealed a significant difference in the correlation strengths between short-delay recall and AHI during REM sleep versus during NREM sleep (z = 2.81, *p = *0.005), demonstrating that REM AHI was more strongly associated with short-delay recall than NREM AHI. Moreover, multiple regression models indicated that REM-NREM AHI (b = -2.38, *p < *0.001), RDI (b = -1.77, *p = *0.02), and ODI (b = -2.02, *p = *0.03) ratios were negatively associated with short-delay recall, both in models featuring *APOE4* status (See Table S16) and parental history of AD as covariates (all *p < *0.03; See Table S17). Thus, individuals with more severe sleep apnea had worse short-delay recall, particularly if OSA was more prevalent during REM sleep as opposed to during NREM sleep.

### Associations between sleep apnea and long-delay recall

**Fig. 3:**
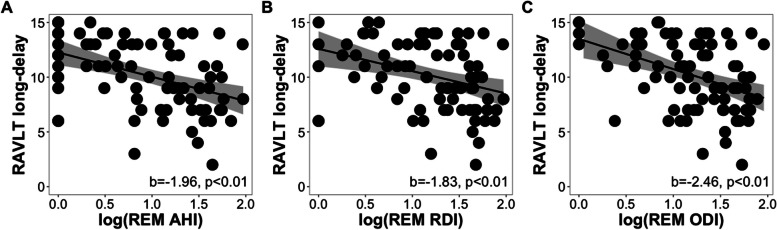
Scatter plots showing the relationships of (**A**) AHI, (**B**) RDI, and (**C**) ODI during REM sleep to RAVLT long-delay scores while controlling for age, sex, time between assessments, years of education, BMI, and *APOE4* status

Total AHI, RDI, and ODI were not significantly associated with long-delay recall (all *p > *0.07). However, REM AHI (b = -1.96, *p = *0.001, FDR-corrected *p = *0.01), REM RDI (b = -1.83, *p = *0.01, FDR-corrected *p = *0.04), and REM ODI (b = -2.46, *p = *0.001, FDR-corrected *p = *0.02) were all significantly associated with worse long-delay recall (Fig. 3A-C). In models with parental history of AD instead of *APOE4* status as the covariate, REM AHI, REM RDI, and REM ODI remained significant predictors (all FDR corrected *p < *0.05). Demonstrating specificity, these same OSA parameters during NREM sleep were not significantly predictive of long-delay recall (NREM AHI (b = 0.07, *p = *0.93), NREM RDI (b = -0.70, *p = *0.93), NREM ODI (b = -0.08, *p = *0.93). Nadir and average oxyhemoglobin desaturation during total sleep, duration spent with < 90% blood oxyhemoglobin saturation across total sleep and in NREM and REM sleep stages, and WASO were also not significant predictors (all *p > *0.12; See Table S18 for full statistical results). Similar insignificant results were found with models with NREM OSA severity predicting long-delay recall that included parental history of AD instead of *APOE4* status (See Table S19 for full statistical results).

**Fig. 4:**
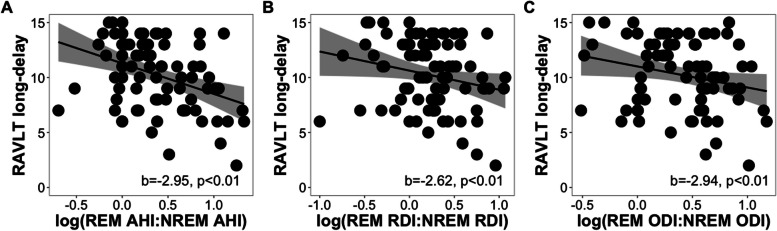
Scatter plots showing the relationships of REM-NREM (**A**) AHI, (**B**) RDI, and (**C**) ODI ratios to RAVLT long-delay scores while controlling for age, sex, time between assessments, years of education, BMI, and *APOE4* status

Steiger’s Z tests revealed significant differences in the correlation strengths between long-delay recall and REM AHI (z = 2.90, *p = *0.004) and REM ODI (z = 2.14, *p = *0.03) versus NREM features, but not in RDI (z = 1.85, *p = *0.07), indicating that the frequency of events and extent of oxyhemoglobin desaturations in REM sleep were more strongly associated with long-delay recall than in NREM sleep. Further, multiple regression models showed that the ratios of REM-NREM AHI (b = -2.95, *p < *0.001), RDI (b = -2.62, *p = *0.001), and ODI (b = -2.94, *p < *0.001) were significantly negatively associated with long-delay recall (Fig. 4A-C; See Table S20). Results from models with parental history of AD in place of *APOE4* status were similar (all *p < *0.003; See Table S21). These results complement our initial findings by demonstrating that that worse verbal memory learning and recall performance were specifically associated with greater OSA severity during REM sleep and not during NREM sleep.

Given the significant associations between REM OSA features and verbal memory, we wanted to account for the possible influence of percentage of REM sleep in these models. We found that even when controlling for percentage of REM sleep, REM AHI, RDI, and ODI, in addition to NREM-REM AHI, NREM-REM RDI, and NREM-REM ODI ratios were still associated with RAVLT measures (all *p > *0.05; See Tables S22 and S23 for full statistical details). Furthermore, we probed whether total number of OSA events during REM sleep or total duration of REM sleep (i.e., the two values that go into calculating OSA indices) may be driving the significant associations between REM OSA features and verbal memory. We found that REM sleep duration was not significantly associated with RAVLT performance (all *p > *0.25; see Tables S24 and S25). In contrast, total amount of apneas and hypopneas in REM sleep was associated with RAVLT total learning, short-delay, and long-delay significantly or on a trend level (all *p < *0.09; See Tables S26 and S27 for full statistical details). Total number of respiratory-related arousals in addition to apneas/hypopneas (the total number of events in RDI) in REM sleep were also related to RAVLT total learning and long-delay recall (ps < 0.05; See Tables S28 and S29 for full statistical details). Lastly, total number of oxyhemoglobin desaturations during REM sleep was associated with total learning and long-delay recall (all *p < *0.05; see Tables S30 and S31 for full statistical details). All models controlled for the same covariates with either *APOE*4 status or parental history of AD. These findings suggest that the impact of OSA during REM sleep on verbal memory is more strongly associated with the OSA-related events themselves rather than their effects on REM sleep duration, per se. As a control analysis, we also analyzed whether periodic limb movement of sleep index (PMLSI) was associated with RAVLT performance. We found that there was no significant association between PLSMI and verbal memory (all *p < *0.06).

### The moderating effects of AD risk factors on verbal memory

**Fig. 5:**
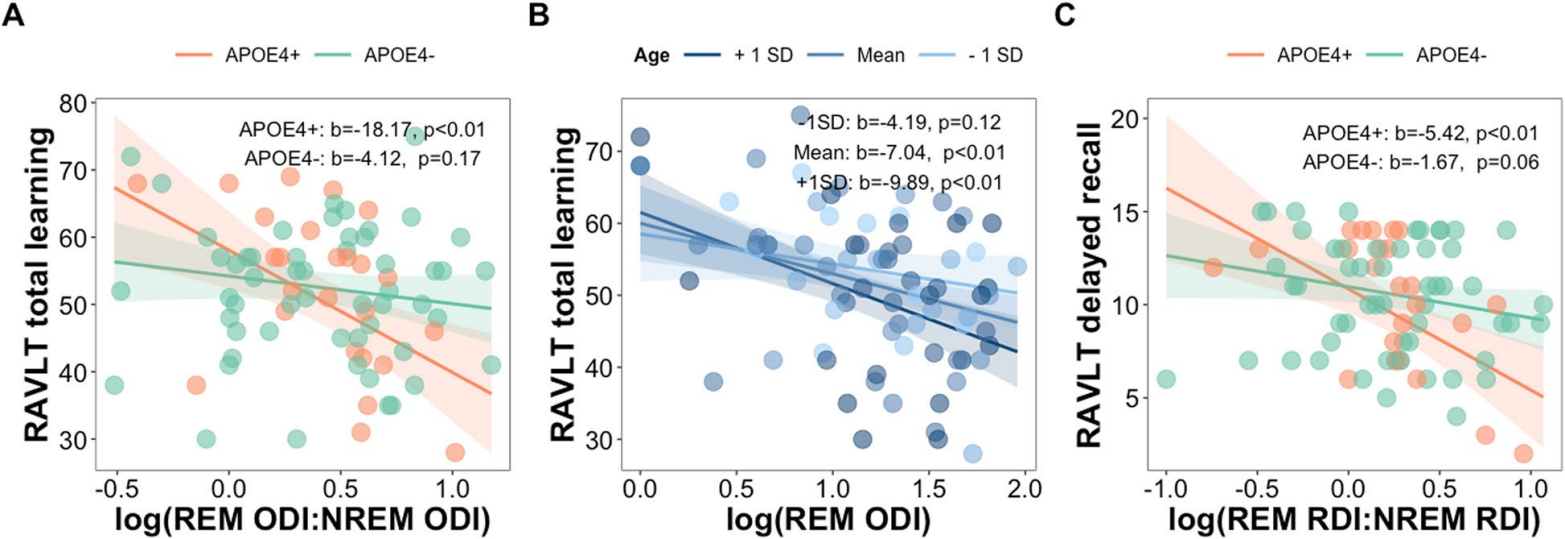
**A** The association between REM-NREM ODI ratio and RAVLT total learning scores was significantly moderated by *APOE4* status. Only the *APOE4* carriers showed that more oxyhemoglobin saturations during REM sleep as opposed to NREM sleep was related to worse learning performance. **B** The association between REM ODI and RAVLT total learning scores was moderated by age. A significant moderating effect was present at the mean age and 1 SD above the mean age (in 80% of the sample). **C** The association between REM-NREM RDI ratio and RAVLT long-delay recall score was moderated by *APOE4* status. A significant moderating effect was observed for only *APOE4* carriers in that, more respiratory events during REM sleep than in NREM sleep was associated with fewer words remembered after a 20-min delay

Next, we examined the moderating influence of sex, age, and genetic and familial risk of AD on the significant relationships between OSA variables (i.e., OSA indices in REM sleep and REM-NREM ratios) and verbal memory. We found that *APOE4* carriers demonstrated a significant association between REM-NREM ODI ratio and total learning (b = -18.17, *p < *0.01) as opposed to non-carriers (b = -4.12, *p = *0.17; Fig. 5A). Full statistical details with all interactions between OSA features and AD risk factors predicting RAVLT total learning (with *APOE4* status as a covariate in the models) are presented in Table S32. In models with parental history of AD as the covariate, age significantly moderated the association between REM ODI and total learning (b = -0.47, *p < *0.05). There were significant effects at the mean age and at 1SD above the mean age (all *p < *0.01; Fig. 5B), with 80% of the sample in the significant moderating range (Figure S1). Full statistical details with all interactions between OSA features and AD risk factors predicting RAVLT total learning (with parental history of AD as a covariate in the models) are presented in Table S33. Taken together, these findings indicate that the negative impact of oxyhemoglobin desaturations during REM sleep (relative to NREM sleep) on RAVLT total learning was more pronounced in those that were *APOE4* carriers and in those aged 60 or older. Further, REM-NREM RDI ratio was significantly associated with long-delay recall in *APOE4* carriers (b = -5.42, *p < *0.01) but not in those without *APOE4* (b = -1.67, *p = *0.06; Fig. 5C). Full statistical details with all interactions between OSA features and AD risk factors predicting long-delay recall (with *APOE4* status as a covariate in the models) are presented in Table S34. There were no significant interactions between OSA factors and AD risk factors on long-delay recall when parental history of AD was a covariate in the models (See Table S35). This suggests that the association between higher REM-NREM RDI and long-delay recall was specific to individuals with genetic risk for AD.

Lastly, we binned participants into the 3 groups based on presence or absence of *APOE4* status and parental history of AD: 1) people with no AD risk factors, 2) people with either *APOE4* status or parental history of AD, and 3) people with both AD risk factors. We then used ANCOVA models to examine interactions between OSA characteristics and AD risk factor groups as it related to verbal memory. In individuals with both AD risk factors, we found that higher REM RDI (b = -14.08, 95%CI:[-21.53, -6.63]), REM-NREM RDI ratio (b = -23.20, 95%CI:[-35.34, -11.06]) and REM-NREM ODI (b = -23.49, 95%CI:[-35.46, -11.51]) were significantly associated with worse total learning (See Figure S2 and Tables S36-S38 for contrast testing). Similarly, in individuals that were both *APOE4* positive and had parental history of AD, higher REM-NREM RDI ratio was significantly associated with lower long-delay recall (b = -8.27, 95%CI:[-12.13, -4.42]; See Figure S3 and Table S39 for contrast testing results). Overall, these findings suggest that more OSA-related events in REM sleep (relative to NREM sleep) strongly impaired word list learning and recall, especially for those that had both parental and genetic risk for AD.

## Discussion

In this study, we assessed the relationships between OSA features and verbal memory performance of a word list, and tested the moderating effects of biological sex, age, *APOE4* status, and parental history of AD on these relationships. We found that greater OSA severity during REM sleep was associated with worse word list learning and delay memory recall in a cohort of cognitively unimpaired middle- and older- aged adults enriched for AD risk. Additionally, more oxyhemoglobin desaturations during REM sleep versus NREM sleep were associated with worse learning performance, specifically in those that were older than 60 years old and *APOE4* carriers. Further, more respiratory events and arousals during REM sleep, as opposed to during NREM sleep, had a greater negative impact on recall performance for those who were *APOE4* carriers. The negative effects of OSA during REM sleep, specifically respiratory disturbances and oxyhemoglobin desaturations, on verbal memory seemed to be most prominent in those that had a parent with AD and was an *APOE4* carrier. Since AD risk factors (e.g., female sex, older age, or genetic or familial risk) were not associated with more severe OSA in this current study, these findings were not simply driven by increased OSA severity in individuals with AD risk factors. Though it is possible this could be related to lower survival from conversion to mild cognitive impairment (MCI) or AD in older adults with AD risk and more severe OSA [9, 21, 68, 69]. That being said, our results support the hypothesis that the memory consequences of OSA are particularly important for cognitively intact older adults with AD risk factors (older age, *APOE4* positivity, and parental history of AD), particularly when OSA events occur during REM sleep.

OSA predominantly expressed in REM sleep is a common condition and REM-sleep related physiological changes lead to increased susceptibility to airway collapse, with longer durations of apneas and hypopneas and more severe oxyhemoglobin desaturations [19, 20, 53, 70]. This is consistent with our findings that demonstrated higher AHI, RDI, and ODI scores during REM sleep relative to NREM sleep and extends the current literature by demonstrating that OSA events in REM sleep were more strongly linked with verbal memory performance than OSA events in NREM sleep. However, future investigations comparing samples enriched for more severe REM or NREM OSA are needed to determine whether it is specifically REM OSA severity that negatively impacts verbal memory performance.

REM OSA may impact verbal memory learning and recall via active disruption of memory processing or through long-term damage to brain structures and brain network function relevant for memory processing during REM sleep. While considerable attention has been given to the role of NREM sleep features in memory processing [71], there is evidence that REM sleep also supports memory. Neuroplastic processes needed for both memory consolidation and forgetting has been observed during REM sleep, in addition, hippocampal replay also occurs during this sleep stage [72, 73]. Further, behaviorally, REM sleep has been linked to both emotional and spatial navigational memory [74]. Metabolic demand is also greater during REM sleep as opposed to wake and NREM sleep, including in memory-relevant regions, such as the MTL [17, 18]. Therefore, OSA events in REM sleep could potentially cause memory deficits through both 1) transient disruptions in cerebral glucose metabolism in memory networks actively supporting memory processing during REM sleep and 2) long term degeneration of memory networks resultant from the presence of repeated hypoxia during high metabolic demand.

Varga and colleagues demonstrated the acute cognitive consequences of REM OSA in which they found that when withdrawing positive airway pressure (PAP) treatment specifically during REM sleep, spatial memory performance was reduced when compared to continued PAP treatment during REM sleep [16]. Although, the impact of withdrawing treatment during NREM sleep was not assessed, these findings indicated that REM OSA could cause transient MTL dysfunction by actively disrupting memory formation and consolidation even prior to neurodegenerative processes.

The effects of intermittent hypoxemia during OSA is a likely contributor to the hippocampal atrophy reported in people with OSA and explains memory impairments observed in OSA [5–9, 13, 14]. A possible mechanism of OSA’s impact on the hippocampus is through the presence of AD pathologies, with evidence supporting that hypoxemia exacerbates expression of both β-amyloid and tau that will in turn cause neurodegeneration and cognitive deficits [75–77]. Another potential mechanism is that hypoxia and sleep fragmentation specifically in REM sleep could accelerate neurodegeneration and cognitive decline via a vascular pathway [54–56, 78, 79]. REM sleep is characterized by increased sympathetic activation, decreased vagal tone, and cardiovascular instability, and REM OSA has been linked to poor cardiovascular health [20, 54–56]. While REM sleep has shown to have high cerebral blood flow in memory-relevant brain areas, REM OSA severity has also been associated with reduced regional cerebral blood flow in those regions [78, 79]. In addition, older adults with cardiovascular risk factors were more likely to have memory deficits [80]. Thus, it is possible that the compounded effects of REM OSA and vascular dysfunction greatly increases oxidative stress, neuroinflammation, blood brain barrier breakdown, and/or endothelial dysfunction causing neurodegenerative-associated memory deficits in older adults [20, 77].

We found that AD risk factors including older age and both parental and genetic risk for AD all exacerbated the effect of OSA severity during REM sleep on word list learning and recall. While it has been reported that the associations between OSA and cognition are weaker in older age, our findings suggested in contrast, that the relationship between oxygen desaturations in REM sleep and verbal memory were actually strongest in older individuals [9]. As our cohort consisted of individuals with undiagnosed OSA and we are unaware of the true age of OSA onset, it is quite possible that some of the older participants may have had untreated OSA longer than the younger participants. We thus cannot discount that our findings may be more related to the consequences of the duration of untreated OSA than age of OSA onset, per se.

While it is remains unclear whether *APOE4* status increases risk for SDB, our findings suggest that *APOE4* carriers may be more vulnerable to the impact of OSA, especially during REM sleep, on memory function. Other studies have reported similar findings in that, in *APOE4* carriers, OSA severity was associated with worse memory and executive function and had increased odds of cognitive decline [25, 81–83]. Furthermore, disrupted sleep and *APOE4* status may synergistically exacerbate expression of hallmark AD pathologies of β-amyloid and tau [84, 85].

The combined effects of parental history of AD and *APOE4* positivity has shown to have strong negative effects on learning and memory [86, 87]. In addition, older age, family history of AD, and *APOE4* status have been linked to a smaller hippocampus and greater accumulation of pathological β-amyloid and tau [88, 89]. Moreover, in a subset of this cohort, we found that increasing age was related to elevated cerebral spinal fluid (CSF) markers of tau phosphorylation and neuroinflammation, which were then associated with impaired sleep-dependent memory [43]. This points to the possibility that accumulation of AD pathologies might be intensified by REM-related OSA leading to poor memory function, with the effects strongest or even just specific to those that are older and with parental and/or genetic risk for AD. Alternatively, REM OSA may contribute to cognitive impairment through cerebrovascular disease, and may be a factor to the common comorbidity of AD and vascular cognitive impairment [90]. Prospective studies will be necessary to investigate whether REM OSA accelerates expression of AD pathologies or promotes cognitive impairment through cerebrovascular dysfunction, or both, as well as why individuals with AD risk factors and OSA may be more cognitively impaired.

We did not find sex-specific effects in the associations between OSA and memory. In this specific cohort, males presented with more severe NREM and REM OSA, and had worse verbal memory performance than females. The lack of a sex effect could be due to a cognitively healthy sample that included females with a less severe OSA presentation. While females have increased risk for AD and present with greater levels of pathological tau in regions associated with AD compared to males, it is possible that the negative effects of OSA on verbal memory performance may be more exaggerated only once women are tau and/or β-amyloid positive, due to the female verbal memory advantage [28, 29, 67, 91, 92]. This verbal memory advantage tends to be diminished when women progress from MCI to AD who present with steeper memory decline than men [30]. In support of this possibility, the average age of this cohort was < 65 years old and in a subsample of 58 participants from this cohort, they were almost entirely β-amyloid and tau negative (based on CSF assessment) [43]. Future studies are needed that combine multimodal neuroimaging, sleep apnea testing, and other cognitive measures, to examine this in more detail. Regardless, it is important to state that despite the cohort being largely β-amyloid and tau negative, AD risk still remained a significant moderator of OSA-memory relationships, indicating that these effects cannot be entirely explained by and may even precede β-amyloid positivity, despite recent findings [26].

Some limitations of this study should be addressed. This was a cross-sectional study that found correlational relationships between OSA characteristics during REM sleep and verbal memory. Longitudinal studies will be necessary to examine how treatment of REM-related OSA would affect memory decline and progression to MCI or AD. Given the study sample size and number of analyses computed, it is possible that the study was underpowered to detect some significant associations. However, the focus of the current study was to contrast the relative strengths of associations between OSA features and memory when events occurred during NREM or REM sleep. While we think these effects are likely robust, it will be important to replicate these findings in a larger study. Further, the memory testing and sleep measurements did not typically occur on the same day. While we controlled for time between measurements, this study does not directly address memory processing that occurs over a night of sleep, but rather informs upon sleep abnormalities and memory associations at the trait level of individual differences. Another limitation is that this study had exclusions of multiple medications that are commonly taken by older adults, which could potentially bias the sample and reduce the generalizability of the results. It is also important to note that this cohort was mostly White (88%) and that these findings may not be generalizable, since racial/ethnic disparities and differences exist for both OSA and AD risk factors and the relationships between these risk factors are not well studied in underrepresented populations [93, 94].

Our findings further support the possibility that OSA could be a modifiable risk factor for AD through its impact on one of the more sensitive markers of AD, impairment in word list learning and recall. A future direction is to extend our analyses to examine OSA’s relationship with other cognitive measures that are impaired in AD, including verbal memory of stories, nonverbal memory, and executive function. This will further elucidate OSA as a contributor to AD and provide further support that treatment may reduce risk for cognitive decline [9]. There is some evidence that continuous positive airway pressure (CPAP) adherence decreases the odds of AD dementia and slowed cognitive decline [95–97]. Importantly, a systematic review reported that PAP treatment adherence only covers mostly the first half of the night, which could potentially leave much of REM sleep OSA untreated, since REM sleep dominates the latter half of the night [98]. It will be critical for future investigations to examine whether more aggressive OSA treatment that covers the entire sleep period would mitigate cognitive impairment and AD risk in individuals with OSA. With growth of the aging population, there is a need for interventions targeting prevention of MCI and AD, and early diagnosis and effective treatment of OSA may be one approach that could reduce risk for neurodegenerative diseases and cognitive dysfunction associated with AD.

## Conclusion

In conclusion, these findings suggest that more severe OSA during REM sleep and more REM OSA events as opposed to NREM OSA events were linked to worse verbal memory performance. This relationship was particularly true for older adults and individuals with a genetic risk for and parental history of AD. This suggests that the negative memory consequences of OSA, specifically when OSA events occur during REM sleep, are particularly impactful in individuals with multiple AD risk factors. The findings emphasize the importance of a thorough OSA screening with sleep recording capable of assessing sleep stage specific expression of OSA, as certain individuals may have high REM AHI, while presenting with a low overall AHI. This is particularly important given that most ambulatory, non-PSG methods for assessing OSA do not include the capacity to assess REM versus NREM sleep specific OSA expression. Without sleep stage characterization of OSA, individuals that are more susceptible to memory decline, especially those with AD risk factors, may miss the opportunity to be referred for comprehensive neurological/neuropsychological evaluation and aggressive OSA treatment that may delay cognitive decline and/or AD onset.

### Supplementary Information

**Supplementary Material 1.**

**Supplementary Material 2.**

## Data Availability

The data are available upon reasonable request and can be obtained by completing a Wisconsin Alzheimer’s Disease Research Center resource request: https://www.adrc.wisc.edu/apply-resources.

## Abbreviations

AD: Alzheimer’s disease
ADRC: Alzheimer’s Disease Research Center
AHI: Apnea–hypopnea Index
ANCOVA: Analysis of covariance
APOE4: Apolipoprotein E ε4
BMI: Body mass index
CPAP: Continuous Positive Airway Pressure
CSF: Cerebral spinal fluid
FDR: False Discovery Rate
hdEEG: High-density electroencephalography
MCI: Mild cognitive impairment
MTL: Medial temporal lobe
NREM: Non-rapid eye movement
ODI: Oxyhemoglobin Desaturation Index
OSA: Obstructive Sleep Apnea
PAP: Positive airway pressure
PLMSI: Periodic leg movements during sleep index
PSG: Polysomnography
RAVLT: Rey Auditory Verbal Learning Test
RDI: Respiratory Disturbance Index
REM: Rapid eye movement
SBD: Sleep Disordered Breathing
TST: Total sleep time
TIB: Time in bed
WASO: Wake after sleep onset
