# Supplementary material for: Older adults at greater risk for Alzheimer’s disease show stronger associations between sleep apnea severity in REM sleep and verbal memory
Source: Alzheimers Res Ther. 2024 May 9;16:102. doi: 10.1186/s13195-024-01446-3 (PMC11080222; doi:10.1186/s13195-024-01446-3)
Supplement: Supplementary file 1 — Supplementary Material 1. [file 13195_2024_1446_MOESM1_ESM.docx]

**SUPPLMENTARY TABLES: Older adults at greater risk for Alzheimer’s disease show stronger associations between sleep apnea severity in REM sleep and verbal memory**

Kitty K. Lui^1,2^, Abhishek Dave^2,3^, Kate E. Sprecher^4-7^, Miranda G. Chappel-Farley^8,9^, Brady A. Riedner^10^, Margo B. Heston^6,7^, Chase E. Taylor^11^, Cynthia M. Carlsson^6,7,12,13^, Ozioma C. Okonkwo^6,7,12,13^, Sanjay Asthana^6,7,12,13^, Sterling C. Johnson^6,7,12,13^, Barbara B. Bendlin^6,7,12,13^, Bryce A. Mander^2,3,9*^, Ruth M. Benca^2,5,9,10,14*^

^1^San Diego State University/University of California San Diego, Joint Doctoral Program in Clinical Psychology, San Diego, CA, USA

^2^Department of Psychiatry and Human Behavior, University of California, Irvine, CA, USA

^3^Department of Cognitive Sciences, University of California, Irvine, CA, USA

^4^Department of Population Health Sciences, University of Wisconsin-Madison, Madison, WI, USA

^5^Neuroscience Training Program, University of Wisconsin-Madison, Madison, WI, USA

^6^Department of Medicine, University of Wisconsin-Madison, Madison, WI, USA

^7^Wisconsin Alzheimer's Disease Research Center, University of Wisconsin-Madison, Madison, WI, USA

^8^Department of Neurobiology and Behavior, University of California, Irvine, CA, USA

^9^Center for the Neurobiology of Learning and Memory, University of California, Irvine, CA, USA

^10^Department of Psychiatry, University of Wisconsin-Madison, Madison, WI, USA

^11^Department of Neuroscience, University of Kentucky, Lexington, KY, USA

^12^Wisconsin Alzheimer’s Institute, Madison, WI, USA

^13^Geriatric Research Education and Clinical Center, Wm. S. Middleton Veterans Hospital, Madison, WI, USA

^14^Department of Psychiatry and Behavioral Medicine, Wake Forest University, Winston-Salem, NC, USA

*Correspondence should be addressed to:

Ruth M. Benca, M.D., Ph.D., rbenca@wakehealth.edu | (336) 716-2911

Bryce A. Mander, Ph.D., [bmander@uci.edu](mailto:bmander@uci.edu) | (949) 824-6742

**Table S1. Uniform Data Set Neuropsychological Battery (n=81)**

|  | **Mean [SD]** |
| --- | --- |
| **Time between PSG and UDS** | 0.02 [0.1] |
| **Time between RAVLT and UDS** | 0.3 [0.9] |
|  |  |
| **Montreal Cognitive Assessment** | 27.6 [2.1] |
|  |  |
| **Craft Story** |  |
| Immediate Recall (verbatim, total units) | 24.4 [5.6] |
| Delay Recall (verbatim, total units) | 21.5 [6.0] |
| Immediate Recall (paraphrase, total units) | 17.4 [3.4] |
| Delay Recall (paraphrase, total units) | 16.6 [3.9] |
|  |  |
| **Benson Complex Figure** |  |
| Immediate Recall | 15.8 [0.9] |
| Delay Recall | 13.2 [2.3] |
|  |  |
| **Multilingual Naming Test** | 30.6 [1.8] |
|  |  |
| **Verbal Fluency: Phonemic Test** |  |
| Letter Fluency (Total F words in 60 s) | 16.4 [4.4] |
| Letter Fluency (Total L words in 60 s) | 15.4 [4.2] |
| Letter Fluency (Total F and L words in 60 s) | 31.8 [7.4] |
| Category Fluency (Vegetable in 60 s) | 16.8 [4.2] |
|  |  |
| **Trail Making Test** |  |
| Trails A (time in s) | 21.8 [8.1] |
| Trails B (time in s) | 55.3 [30.0] |

UDS= Uniform Data Set; PSG= polysomnography; RAVLT=Rey Auditory Verbal

Learning Test; *Neuropsychological battery missing from one participant.*

**Table S2. Sex differences on OSA characteristics**

|  | Female (n=49) | | Male (n=32) |  |
| --- | --- | --- | --- | --- |
|  | Mean [SD] | Mean [SD] | | *p* |
| log(TST AHI) | 0.51 [0.43] | | 0.93 [0.50] | **<0.001*** |
| log(REM AHI) | 0.78 [0.61] | | 1.13 [0.57] | **0.018*** |
| log(NREM AHI) | 0.35 [0.37] | | 0.82 [0.55] | **<0.001*** |
| log(TST RDI) | 0.87 [0.42] | | 1.25 [0.45] | **<0.001*** |
| log(REM RDI) | 1.05 [0.54] | | 1.33 [0.48] | **0.021*** |
| log(NREM RDI) | 0.76 [0.42] | | 1.19 [0.50] | **<0.001*** |
| log(TST ODI) | 0.80 [0.38] | | 1.10 [0.48] | **0.002*** |
| log(REM ODI) | 1.12 [0.49] | | 1.12 [0.48] | 0.118 |
| log(NREM ODI) | 0.63 [0.40] | | 1.00 [0.52] | **<0.001*** |
| Nadir Blood Oxyhemoglobin saturation (%) | 87.25 [6.29] | | 83.13 [9.62] | **0.025*** |
| Mean Blood Oxyhemoglobin saturation (%) | 95.25 [1.67] | | 94.06 [1.48] | **0.001*** |
| TST Duration <90% Blood Oxyhemoglobin saturation (mins) | 1.68 [3.62] | | 8.50 [13.08] | **0.001*** |
| REM Duration <90% Blood Oxyhemoglobin saturation (mins) | 0.78 [2.07] | | 3.44 [6.54] | **0.003*** |
| NREM Duration <90% Blood Oxyhemoglobin saturation (mins) | 0.90 [1.89] | | 5.07 [10.86] | **0.002*** |
| WASO (mins) | 79.95 [57.12] | | 104.34 [61.47] | **0.043*** |
| log(REM AHI: NREM AHI) | 0.43 [0.44] | | 0.32 [0.43] | 0.392 |
| log(REM RDI: NREM RDI) | 0.29 [0.42] | | 0.14 [0.37] | 0.115 |
| log(REM ODI: NREM ODI) | 0.49 [0.38] | | 0.29 [0.36] | **0.017*** |

TST: total sleep time; AHI: apnea-hypopnea index; REM: rapid eye movement;

NREM: non-rapid eye movement; RDI: respiratory disturbance index;

ODI: oxygen desaturation index; WASO: Wake after sleep onset

*p<0.05 significant difference indicated by independent sample t-test

**Table S3. Correlation between age and OSA characteristics**

|  | Age | |
| --- | --- | --- |
|  | τ | *p* |
| log(TST AHI) | 0.043 | 0.576 |
| log(REM AHI) | 0.054 | 0.481 |
| log(NREM AHI) | 0.067 | 0.388 |
| log(TST RDI) | 0.072 | 0.342 |
| log(REM RDI) | 0.054 | 0.480 |
| log(NREM RDI) | 0.093 | 0.220 |
| log(TST ODI) | 0.096 | 0.208 |
| log(REM ODI) | 0.074 | 0.332 |
| log(NREM ODI) | 0.121 | 0.110 |
| Nadir Blood Oxyhemoglobin saturation (%) | -0.089 | 0.249 |
| Mean Blood Oxyhemoglobin saturation (%) | -0.129 | 0.111 |
| TST Duration <90% Blood Oxyhemoglobin saturation (mins) | 0.077 | 0.335 |
| REM Duration <90% Blood Oxyhemoglobin saturation (mins) | 0.048 | 0.553 |
| NREM Duration <90% Blood Oxyhemoglobin saturation (mins) | 0.111 | 0.177 |
| WASO | 0.142 | 0.062 |
| log(REM AHI:NREM AHI) | -0.011 | 0.890 |
| log(REM RDI: NREM RDI) | -0.049 | 0.514 |
| log(REM ODI: NREM ODI) | -0.048 | 0.552 |

τ: Kendall rank correlation coefficient; TST: total sleep time;

AHI: apnea-hypopnea index; REM: rapid eye movement;

NREM: non-rapid eye movement; RDI: respiratory disturbance index;

ODI: oxygen desaturation index; WASO: Wake after sleep onset

*p<0.05 significant association indicated by Kendall rank correlation

**Table S4. *APOE*4 carrier status differences on OSA characteristics**

|  | *APOE4*- (n=54) | *APOE4*+ (n=26) |  |
| --- | --- | --- | --- |
|  | Mean [SD] | Mean [SD] | *p* |
| log(TST AHI) | 0.76 [0.50] | 0.50 [0.47] | **0.034*** |
| log(REM AHI) | 1.00 [0.58] | 0.71 [0.63] | **0.044*** |
| log(NREM AHI) | 0.60 [0.52] | 0.38 [0.42] | **0.044*** |
| log(TST RDI) | 1.09 [0.44] | 0.86 [0.50] | **0.041*** |
| log(REM RDI) | 1.22 [0.52] | 1.02 [0.55] | 0.097 |
| log(NREM RDI) | 1.00 [0.48] | 0.79 [0.52] | 0.111 |
| log(TST ODI) | 0.99 [0.43] | 0.74 [0.44] | **0.018*** |
| log(REM ODI) | 1.26 [0.45] | 1.02 [0.53] | 0.052 |
| log(NREM ODI) | 0.86 [0.49] | 0.61 [0.43] | **0.037*** |
| Nadir Blood Oxygenation (%) | 85.48 [7.13] | 86.31 [9.55] | 0.155 |
| Mean Blood Oxygenation (%) | 94.70 [1.73] | 94.96 [1.63] | 0.316 |
| TST Duration <90% Blood Oxyhemoglobin saturation (mins) | 5.34 [10.28] | 2.45 [6.58] | 0.231 |
| REM Duration <90% Blood Oxyhemoglobin saturation (mins) | 2.30 [5.28] | 0.84 [2.47] | 0.136 |
| NREM Duration <90% Blood Oxyhemoglobin saturation (mins) | 3.04 [8.04] | 1.62 [5.24] | 0.253 |
| WASO (mins) | 89.00 [60.17] | 90.23 [61.02] | 0.857 |
| log(REM AHI: NREM AHI) | 0.40 [0.47] | 0.33 [0.35] | 0.817 |
| log(REM RDI: NREM RDI) | 0.22 [0.43] | 0.23 [0.35] | 0.918 |
| log(REM ODI: NREM ODI) | 0.40 [0.41] | 0.41 [0.32] | 0.925 |

*APOE* = apolipoprotein E; TST: total sleep time; AHI: apnea-hypopnea index;

REM: rapid eye movement; NREM: non-rapid eye movement;

RDI: respiratory disturbance index; ODI: oxygen desaturation index

*p<0.05 significant difference indicated by independent sample t-test

**Table S5. Parental AD history status differences on OSA characteristics**

|  | Parental AD History- (n=25) | Parental AD History+ (n=56) |  |
| --- | --- | --- | --- |
|  | Mean [SD] | Mean [SD] | *p* |
| log(TST AHI) | 0.66 [0.56] | 0.68 [0.48] | 0.866 |
| log(REM AHI) | 0.93 [0.64] | 0.90 [0.60] | 0.678 |
| log(NREM AHI) | 0.51 [0.57] | 0.54 [0.47] | 0.463 |
| log(TST RDI) | 0.99 [0.51] | 1.02 [0.45] | 0.854 |
| log(REM RDI) | 1.13 [0.60] | 1.16 [0.51] | 0.886 |
| log(NREM RDI) | 0.90 [0.55] | 0.95 [0.48] | 0.673 |
| log(TST ODI) | 0.93 [0.47] | 0.90 [0.44] | 0.620 |
| log(REM ODI) | 1.21 [0.47] | 1.17 [0.50] | 0.609 |
| log(NREM ODI) | 0.79 [0.52] | 0.77 [0.47] | 0.760 |
| Nadir Blood Oxyhemoglobin saturation (%) | 86.29 [6.13] | 85.52 [8.64] | 0.878 |
| Mean Blood Oxyhemoglobin saturation (%) | 94.83 [2.26] | 94.77 [1.41] | 0.771 |
| TST Duration <90% Blood Oxyhemoglobin saturation (mins) | 5.78 [10.79] | 3.81 [8.62] | 0.979 |
| REM Duration <90% Blood Oxyhemoglobin saturation (mins) | 2.06 [5.63] | 1.73 [4.13] | 0.712 |
| NREM Duration <90% Blood Oxyhemoglobin saturation (mins) | 3.72 [7.82] | 2.09 [7.01] | 0.671 |
| WASO (mins) | 95.54 [64.87] | 86.77 [58.30] | 0.606 |
| log(REM AHI:NREM AHI) | 0.42 [0.41] | 0.35 [0.44] | 0.364 |
| log(REM RDI: NREM RDI) | 0.24 [0.24] | 0.22 [0.37] | 0.664 |
| log(REM ODI: NREM ODI) | 0.42 [0.40] | 0.40 [0.38] | 0.782 |

AD: Alzheimer’s disease; TST: total sleep time; AHI: apnea-hypopnea index;

REM: rapid eye movement; NREM: non-rapid eye movement;

RDI: respiratory disturbance index; ODI: oxygen desaturation index

WASO: Wake After Sleep Onset

*p<0.05 significant difference indicated by independent sample t-test

**Table S6. Sex differences on RAVLT scores**

|  | Female (n=50) | | Male (n=32) |  |
| --- | --- | --- | --- | --- |
|  | Mean [SD] | Mean [SD] | | *p* |
| Total Learning | 54.22 [10.18] | | 47.66 [9.79] | **0.005*** |
| Short-Delay Recall | 11.22 [2.96] | | 10.10 [2.70] | **0.037*** |
| Long-Delay Recall | 10.94 [3.24] | | 9.13 [2.83] | **0.004*** |

RAVLT: Rey Auditory Verbal Learning Test

*p<0.05 significant difference indicated by independent sample t-test

**Table S7. Correlation between age and RAVLT scores**

|  | Age | |
| --- | --- | --- |
|  | τ | *p* |
| Total Learning | -0.119 | 0.123 |
| Short-Delay Recall | -0.064 | 0.421 |
| Long-Delay Recall | -0.102 | 0.196 |

RAVLT: Rey Auditory Verbal Learning Test

τ: Kendall rank correlation coefficient;

*p<0.05 significant association indicated by Kendall rank correlation

**Table S8. *APOE*4 carrier status differences on RAVLT scores**

|  | *APOE4*- (n=54) | *APOE4*+ (n=26) |  |
| --- | --- | --- | --- |
|  | Mean [SD] | Mean [SD] | *p* |
| Total Learning | 51.72 [9.97] | 51.85 [11.66] | 0.961 |
| Short-Delay Recall | 10.96 [2.71] | 10.42 [3.33] | 0.620 |
| Long-Delay Recall | 10.35 [3.05] | 10.00 [3.51] | 0.800 |

*APOE* = apolipoprotein E; RAVLT: Rey Auditory Verbal Learning Test

*p<0.05 significant difference indicated by independent sample t-test

**Table S9. Parental AD risk status differences on RAVLT scores**

|  | Parental AD Risk- (n=25) | Parental AD Risk+ (n=56) |  |
| --- | --- | --- | --- |
|  | Mean [SD] | Mean [SD] | *p* |
| Total Learning | 45.36[8.82] | 54.43 [10.00] | **<0.001*** |
| Short-Delay Recall | 9.54 [2.38] | 11.32 [2.96] | **0.004*** |
| Long-Delay Recall | 8.75 [2.40] | 10.88 [3.29] | **0.003*** |

AD: Alzheimer’s disease; OSA: Obstructive Sleep Apnea

*p<0.05 significant difference indicated by independent sample t-test

**Table S10. Multiple regression model results of OSA features (predictors) on RAVLT total learning (*APOE4* controlled for in the models)**

| **Predictor** | **Unstandardized b ± SEM** | **Predictor (p [95%CI])** |
| --- | --- | --- |
| **log(TST AHI)** | -4.474±2.615 | 0.091 [-9.688, 0.740] |
| **log(REM AHI)** | -4.835±1.913 | **0.014* [-8.650, -1.020]** |
| **log(NREM AHI)** | -1.316±2.701 | 0.628 [-6.701, 4.069] |
| **log(TST RDI)** | -3.702±2.637 | 0.165 [-8.961, 1.557] |
| **log(REM RDI)** | -5.650±2.086 | **0.008* [-9.809, -1.490]** |
| **log(NREM RDI)** | -1.474±2.499 | 0.557 [-6.458, 3.510] |
| **log(TST ODI)** | -5.173±3.043 | 0.094 [-11.240, 0.895] |
| **log(REM ODI)** | -7.909±2.350 | **0.001* [-12.596, 3.223]** |
| **log(NREM ODI)** | -2.406±2.863 | 0.404 [-8.115, 3.304] |
| **Nadir Blood**  **Oxyhemoglobin saturation** | 0.064±0.139 | 0.645 [-0.212, 0.340] |
| **Mean Blood**  **Oxyhemoglobin saturation** | -0.207±0.673 | 0.759 [-1.548, 1.134] |
| **TST Duration of <90%**  **Blood Oxyhemoglobin saturation** | 0.038±0.134 | 0.779 [-0.230, 0.306] |
| **REM Duration <90%**  **Blood Oxyhemoglobin saturation** | 0.203±0.246 | 0.411 [-0.287, 0.694] |
| **NREM Duration <90%**  **Blood Oxyhemoglobin saturation** | -0.034±0.167 | 0.837 [-0.367, 0.298] |
| **WASO** | -6.705×10^-4^±0.018 | 0.971 [-0.037, 0.036] |

*****p<0.05 significant predictor indicated by multiple regression model

**Table S11. Multiple regression model results of OSA features (predictors) on RAVLT total learning (parental history of AD controlled for in the models)**

| **Predictor** | **Unstandardized b ± SEM** | **Predictor p [95%CI]** |
| --- | --- | --- |
| **log(TST AHI)** | -4.342±2.368 | 0.071 [-9.062, 0.378] |
| **log(REM AHI)** | -4.502±1.754 | **0.012* [-7.999, -1.005]** |
| **log(NREM AHI)** | -1.623±2.449 | 0.510 [-6.506, 3.260] |
| **log(TST RDI)** | -3.791±2.409 | 0.120 [-8.593,1.010] |
| **log(REM RDI)** | -5.451±1.920 | **0.006* [-9.278, -1.624]** |
| **log(NREM RDI)** | -1.868±2.293 | 0.418 [-6.439, 2.702] |
| **log(TST ODI)** | -5.028±2.774 | 0.074 [-10.558,0.502] |
| **log(REM ODI)** | -7.572±2.158 | **<.001* [-11.875, -3.269]** |
| **log(NREM ODI)** | -2.650±2.628 | 0.317 [-7.889, 2.589] |
| **Nadir Blood**  **Oxyhemoglobin saturation** | 0.096±0.130 | 0.462 [-0.163, 0.356] |
| **Mean Blood**  **Oxyhemoglobin saturation** | -0.055±0.634 | 0.931 [-1.320, 1.209] |
| **TST Duration of <90%**  **Blood Oxyhemoglobin saturation** | 0.049±0.123 | 0.694 [-0.197, 0.294] |
| **REM Duration <90%**  **Blood Oxyhemoglobin saturation** | 0.217±0.229 | 0.345 [-0.239, 0.674] |
| **NREM Duration <90%**  **Blood Oxyhemoglobin saturation** | -0.021±0.154 | 0.890 [-0.329, 0.286] |
| **WASO** | 0.001±0.017 | 0.946 [-0.033, 0.036] |

p<0.05 significant predictor indicated by multiple regression model

**Table S12. Multiple regression model results of REM-NREM ratios (predictors) on RAVLT total learning (*APOE4* controlled for in the models)**

| **Predictor** | **Unstandardized b ± SEM** | **Predictor p [95%CI]** |
| --- | --- | --- |
| **log(REM AHI:NREM AHI)** | -6.073±2.316 | **0.011* [-10.691, -1.454]** |
| **log(REM RDI:NREM RDI)** | -6.654±2.538 | **0.011* [-11.715, -1.593]** |
| **log(REM ODI:NREM ODI)** | -7.419±2.661 | **0.007* [-12.725, -2.113]** |

p<0.05 significant predictor indicated by multiple regression model

**Table S13. Multiple regression model results of REM-NREM ratios (predictors) on RAVLT total learning (parental history of AD controlled for in the models)**

| **Predictor** | **Unstandardized b ± SEM** | **Predictor p [95%CI]** |
| --- | --- | --- |
| **log(REM AHI:NREM AHI)** | -5.439±2.160 | **0.014* [-9.745, -1.133]** |
| **log(REM RDI:NREM RDI)** | -6.019±2.377 | **0.014* [-10.759, -1.280]** |
| **log(REM ODI:NREM ODI)** | -7.007±2.501 | **0.007* [-11.993, -2.021]** |

p<0.05 significant predictor indicated by multiple regression model

**Table S14. Multiple regression model results of OSA features (predictors) on RAVLT short-delay (*APOE4* controlled for in the models)**

| **Predictor** | **Unstandardized b ± SEM** | **Predictor (p [95%CI])** |
| --- | --- | --- |
| **log(TST AHI)** | -0.837±0.751 | 0.269 [-2.334, 0.660] |
| **log(REM AHI)** | -1.375±0.543 | **0.014* [-2.457, -0.292]** |
| **log(NREM AHI)** | 0.443±0.766 | 0.565 [-1.08, 1.970] |
| **log(TST RDI)** | -0.441±0.757 | 0.562 [-1.951, 1.068] |
| **log(REM RDI)** | -1.094±0.608 | 0.076 **[**-2.307**,** 0.119**]** |
| **log(NREM RDI)** | 0.144±0.711 | 0.840 [-1.273, 1.562] |
| **log(TST ODI)** | -0.798±0.695 | 0.365 [-2.545, 0.949] |
| **log(REM ODI)** | -1.541±2.350 | **0.030* [-2.926, -0.156]** |
| **log(NREM ODI)** | 0.134±0.817 | 0.870 [-1.494, 1.763] |
| **Nadir Blood**  **Oxyhemoglobin saturation** | -0.002±0.039 | 0.953 [-0.081, 0.076] |
| **Mean Blood**  **Oxyhemoglobin saturation** | 0.008±0.191 | 0.969 [-0.373, 0.388] |
| **TST Duration of <90%**  **Blood Oxyhemoglobin saturation** | -0.016±0.038 | 0. 675 [-0.092, 0.060] |
| **REM Duration <90%**  **Blood Oxyhemoglobin saturation** | 0.020±0.070 | 0.772 [-0.119, 0.160] |
| **NREM Duration <90%**  **Blood Oxyhemoglobin saturation** | -0.034±0.047 | 0.476 [-0.128, 0.060] |
| **WASO** | -0.003±0.005 | 0.622 [-0.013, 0.008] |

p<0.05 significant predictor indicated by multiple regression model

**Table S15. Multiple regression model results of OSA features (predictors) on RAVLT short-delay (parental history of AD controlled for in the models)**

| **Predictor** | **Unstandardized b ± SEM** | **Predictor p [95%CI]** |
| --- | --- | --- |
| **log(TST AHI)** | -0.547±0.727 | 0.454 [-1.996, 0.902] |
| **log(REM AHI)** | -1.128±0.536 | **0.039* [-2.196, -0.060]** |
| **log(NREM AHI)** | 0.574±0.737 | 0.438 [-0.894, 2.043] |
| **log(TST RDI)** | -0.234±0.737 | 0.752 [-1.704, 1.236] |
| **log(REM RDI)** | -0.898±0.600 | 0.139 [-2.095, 0.299] |
| **log(NREM RDI)** | 0.244±0.693 | 0.726 [-1.138, 1.625] |
| **log(TST ODI)** | -0.492±0.852 | 0.566 [-2.191, 1.207] |
| **log(REM ODI)** | -1.291±0.687 | 0.064 [-2.660, -0.078] |
| **log(NREM ODI)** | 0.314±0.796 | 0. 694 [-1.273, 1.901] |
| **Nadir Blood**  **Oxyhemoglobin saturation** | 0.003±0.039 | 0.947 [-0.076, 0.081] |
| **Mean Blood**  **Oxyhemoglobin saturation** | 0.011±0.191 | 0.955 [-0.370, 0.392] |
| **TST Duration of <90%**  **Blood Oxyhemoglobin saturation** | -0.006±0.037 | 0.881 [-0.080, 0.068] |
| **REM Duration <90%**  **Blood Oxyhemoglobin saturation** | 0.031±0.069 | 0.652 [-0.107, 0.107] |
| **NREM Duration <90%**  **Blood Oxyhemoglobin saturation** | -0.023±0.046 | 0.627 [-0.115, 0.070] |
| **WASO** | -0.003±0.005 | 0.611 [-0.013, 0.008] |

p<0.05 significant predictor indicated by multiple regression model

**Table S16. Multiple regression model results of REM-NREM ratios (predictors) on RAVLT short-delay (*APOE4* controlled for in the models)**

| **Predictor** | **Unstandardized b ± SEM** | **Predictor p [95%CI]** |
| --- | --- | --- |
| **log(REM AHI:NREM AHI)** | -2.383±0.628 | **<.001* [-3.635, -1.132]** |
| **log(REM RDI:NREM RDI)** | -1.772±0.725 | **0.017* [-3.216, -0.327]** |
| **log(REM ODI:NREM ODI)** | -2.018±0.759 | **0.010* [-3.531, -0.505]** |

p<0.05 significant predictor indicated by multiple regression model

**Table S17. Multiple regression model results of REM-NREM ratios (predictors) on RAVLT short-delay (parental history of AD controlled for in the models)**

| **Predictor** | **Unstandardized b ± SEM** | **Predictor p [95%CI]** |
| --- | --- | --- |
| **log(REM AHI:NREM AHI)** | -2.188±0.628 | **<.001* [-3.439, -0.937]** |
| **log(REM RDI:NREM RDI)** | -1.631±0.722 | **0.027* [-3.071, -0.192]** |
| **log(REM ODI:NREM ODI)** | -1.953±0.759 | **0.012* [-3.467, -0.440]** |

p<0.05 significant predictor indicated by multiple regression model

**Table S18. Multiple regression model results of OSA features (predictors) on RAVLT long-delay (*APOE4* controlled for in the models)**

| **Predictor** | **Unstandardized**  **b ± SEM** | **Predictor p [95%CI]** |
| --- | --- | --- |
| **log(TST AHI)** | -1.490±0.823 | 0.075 [-3.132, 0.152] |
| **log(REM AHI)** | -1.957±0.586 | **0.001* [-3.126, -0.789]** |
| **log(NREM AHI)** | 0.072±0.854 | 0.933 [-1.631, 1.774] |
| **log(TST RDI)** | -0.889±0.837 | 0.292 [-2.559, 0.781] |
| **log(REM RDI)** | -1.834±0.657 | **0.007* [-3.143, -0.525]** |
| **log(NREM RDI)** | -0.070±0.791 | 0.930 [-1.647, 1.507] |
| **log(TST ODI)** | -1.309±0.967 | 0.180 [-3.238, 0.620] |
| **log(REM ODI)** | -2.458±0.744 | **0.001* [-3.940, -0.975]** |
| **log(NREM ODI)** | -0.075±0.908 | 0.934 [-1.886, 1.736] |
| **Nadir Blood**  **Oxyhemoglobin saturation** | 0.020±0.044 | 0.656 [-0.068, 0.107] |
| **Mean Blood**  **Oxyhemoglobin saturation** | 0.133±0.212 | 0.532 [-0.289, 0.555] |
| **TST Duration of <90%**  **Blood Oxyhemoglobin saturation** | -0.009±0.042 | 0.829 [-0.094, 0.075] |
| **REM Duration <90%**  **Blood Oxyhemoglobin saturation** | -0.009±0.078 | 0.910 [-0.164, 0.147] |
| **NREM Duration <90%**  **Blood Oxyhemoglobin saturation** | -0.010±0.053 | 0.853 [-0.115, 0.095] |
| **WASO** | -0.009±0.006 | 0.121 [-0.020, 0.002] |

p<0.05 significant predictor indicated by multiple regression model

**Table S19. Multiple regression model results of OSA features (predictors) on RAVLT long-delay (parental history of AD controlled for in the models)**

| **Predictor** | **Unstandardized**  **b ± SEM** | **Predictor p [95%CI]** |
| --- | --- | --- |
| **log(TST AHI)** | -1.257±0.785 | 0.114 [-2.821, 0.308] |
| **log(REM AHI)** | -1.722±0.569 | **0.003* [-2.857, -0.588]** |
| **log(NREM AHI)** | 0.574±0.737 | 0.438 [-0.894, 2.043] |
| **log(TST RDI)** | -0.761±0.803 | 0.346 [-2.361, 0.839] |
| **log(REM RDI)** | -1.659±0.638 | **0.011* [-2.932, -0.387]** |
| **log(NREM RDI)** | -0.076±0.759 | 0.920 [-1.590, 1.438] |
| **log(TST ODI)** | -1.070±0.927 | 0.252 [-2.917, 0.777] |
| **log(REM ODI)** | -2.220±0.724 | **0.003* [-3.663, -0.776]** |
| **log(NREM ODI)** | 0.009±0.873 | 0. 992 [-1.731, 1.748] |
| **Nadir Blood**  **Oxyhemoglobin saturation** | 0.025±0.043 | 0.560 [0.060, 0.111] |
| **Mean Blood**  **Oxyhemoglobin saturation** | 0.150±0.208 | 0.474 [-0.266, 0.565] |
| **TST Duration of <90%**  **Blood Oxyhemoglobin saturation** | -0.003±0.041 | 0.949 [-0.084, 0.078] |
| **REM Duration <90%**  **Blood Oxyhemoglobin saturation** | -0.001±0.076 | 0.988 [-0.153, 0.150] |
| **NREM Duration <90%**  **Blood Oxyhemoglobin saturation** | -0.003±0.051 | 0.949 [-0.105, 0.098] |
| **WASO** | -0.009±0.006 | 0.120 [-0.020, 0.002] |

p<0.05 significant predictor indicated by multiple regression model

**Table S20. Multiple regression model results of REM-NREM ratios (predictors) on RAVLT long-delay (*APOE4* controlled for in the models)**

| **Predictor** | **Unstandardized b ± SEM** | **Predictor p [95%CI]** |
| --- | --- | --- |
| **log(REM AHI:NREM AHI)** | -2.945±0.681 | **<.001* [-4.303, -1.586]** |
| **log(REM RDI:NREM RDI)** | -2.620±0.779 | **0.001* [-4.174, -1.066]** |
| **log(REM ODI:NREM ODI)** | -2.943±0.813 | **<.001* [-4.564, -1.322]** |

p<0.05 significant predictor indicated by multiple regression model

**Table S21. Multiple regression model results of REM-NREM ratios (predictors) on RAVLT long-delay (parental history of AD controlled for in the models)**

| **Predictor** | **Unstandardized b ± SEM** | **Predictor p [95%CI]** |
| --- | --- | --- |
| **log(REM AHI:NREM AHI)** | -2.684±0.672 | **<.001* [-4.024, -1.344]** |
| **log(REM RDI:NREM RDI)** | -2.404±0.767 | **0.002* [-3.934, -0.875]** |
| **log(REM ODI:NREM ODI)** | -2.832±0.802 | **< .001* [-4.431, -1.234]** |

p<0.05 significant predictor indicated by multiple regression model

**Table S22. Multiple regression model results of REM OSA characteristics and REM-NREM ratios on RAVLT total learning while controlling for percentage of REM sleep (*APOE4* status controlled for in these models)**

| **Predictor** | **Unstandardized b ± SEM** | **Predictor p [95%CI]** |
| --- | --- | --- |
| **log(REM AHI)** | -4.863±1.896 | **0.012* [-8.645, -1.081]** |
| **log(REM RDI)** | -5.342±2.098 | **0.013* [-9.527, -1.157]** |
| **log(REM ODI)** | -7.871±2.330 | **0.001* [-12.518, -3.223]** |
| **log(REM AHI:NREM AHI)** | -3.142±0.668 | **<.001* [-4.475, -1.810]** |
| **log(REM RDI:NREM RDI)** | -6.888±2.512 | **0.008* [-11.898, -1.879]** |
| **log(REM ODI:NREM ODI)** | -7.524±2.634 | **0.006* [-12.779, -2.270]** |

p<0.05 significant predictor indicated by multiple regression model

**Table S23. Multiple regression model results of REM OSA characteristics and REM-NREM ratios on RAVLT total learning while controlling for percentage of REM sleep (parental history of AD controlled for in these models)**

| **Predictor** | **Unstandardized b ± SEM** | | **Predictor p [95%CI]** |
| --- | --- | --- | --- |
| **log(REM AHI)** | -4.478±1.754 | **0.013* [-7.975, -0.982]** | |
| **log(REM RDI)** | -5.262±1.949 | **0.009* [-9.149, -1.375]** | |
| **log(REM ODI)** | -7.506±2.160 | **<.001* [-11.812, -3.199]** | |
| **log(REM AHI:NREM AHI)** | -5.941±2.169 | **0.008* [-10.265, -1.616]** | |
| **log(REM RDI:NREM RDI)** | -6.278±2.376 | **0.010* [-11.015, -1.541]** | |
| **log(REM ODI:NREM ODI)** | -7.137±2.496 | **0.006* [-12.114, -2.159]** | |

p<0.05 significant predictor indicated by multiple regression model

**Table S24: Multiple regression models results of REM sleep duration (hours) on RAVLT performance while controlling for *APOE4* status**

| **Predictor** | **Outcome** | **Unstandardized b**  **± SEM** | **Predictor p [95%CI]** |
| --- | --- | --- | --- |
| REM sleep duration **→** | Total learning | 2.575±2.288 | 0.264 [-1.988, 7.137] |
| REM sleep duration **→** | Short-delay | 0.392±0.654 | 0.551 [-0.912, 1.695] |
| REM sleep duration **→** | Long-delay | -0.826±0.722 | 0.257 [-0.614, 2.266] |

p<0.05 significant predictor indicated by multiple regression model

**Table S25: Multiple regression models results of REM sleep duration (hours) on RAVLT performance while controlling for parental history of AD**

| **Predictor** | **Outcome** | **Unstandardized b**  **± SEM** | **Predictor p [95%CI]** |
| --- | --- | --- | --- |
| REM sleep duration **→** | Total learning | 1.638±2.169 | 0.452 [-2.685, 5.961] |
| REM sleep duration **→** | Short-delay | 0.200±0.655 | 0.761 [-1.106, 1.507] |
| REM sleep duration **→** | Long-delay | 0.618±0.714 | 0.390 [-0.806, 2.041] |

p<0.05 significant predictor indicated by multiple regression model

**Table S26: Multiple regression models results of total number of apneas and hypopneas in REM sleep on RAVLT performance while controlling for *APOE4* status**

| **Predictor** | **Outcome** | **Unstandardized b ± SEM** | **Predictor p [95%CI]** |
| --- | --- | --- | --- |
| log(REM apneas/hypopneas) **→** | Total learning | -3.317±1.907 | 0.086 [-7.120, 0.485] |
| log(REM apneas/hypopneas) **→** | Short-delay | -1.096±0.537 | **0.045* [-2.168, -0.025]** |
| log(REM apneas/hypopneas) **→** | Long-delay | -1.548±0.587 | **0.010* [-2.717, -0.378]** |

p<0.05 significant predictor indicated by multiple regression model

**Table S27: Multiple regression models results of total number of apneas and hypopneas in REM sleep on RAVLT performance while controlling for parental history of AD**

| **Predictor** | **Outcome** | **Unstandardized**  **b ± SEM** | **Predictor p [95%CI]** |
| --- | --- | --- | --- |
| log(REM apneas/hypopneas) **→** | Total learning | -3.542±1.764 | **0.048* [-7.059, -0.026]** |
| log(REM apneas/hypopneas) **→** | Short-delay | -0.995±0.533 | 0.066 [-2.058, 0.068] |
| log(REM apneas/hypopneas) **→** | Long-delay | -1.478±0.572 | **0.012* [-2.618, -0.339]** |

p<0.05 significant predictor indicated by multiple regression model

**Table S28: Multiple regression models results of total number of respiratory disturbances (apneas/hypopneas and respiratory-related arousals) in REM sleep on RAVLT performance while controlling for *APOE4* status**

| **Predictor** | **Outcome** | **Unstandardized b ± SEM** | **Predictor p [95%CI]** |
| --- | --- | --- | --- |
| log(REM  respiratory disturbances) **→** | Total learning | -3.691±2.062 | 0.078 [-7.803, 0.421] |
| log(REM  respiratory disturbances) **→** | Short-delay | -0.839±0.590 | 0.159 [-2.016**,** 0.337] |
| log(REM  respiratory disturbances) **→** | Long-delay | -1.325±0.647 | **0.044* [-2.614, -0.035]** |

p<0.05 significant predictor indicated by multiple regression model

**Table S29: Multiple regression models results of total number of respiratory disturbances (apneas/hypopneas and respiratory-related arousals) in REM sleep on RAVLT performance while controlling for parental history of AD**

| **Predictor** | **Outcome** | **Unstandardized**  **b ± SEM** | **Predictor p [95%CI]** |
| --- | --- | --- | --- |
| log(REM  respiratory disturbances) **→** | Total learning | -3.970±1.905 | **0.041* [-7.768, -0.172**] |
| log(REM  respiratory disturbances) **→** | Short-delay | -0.734±0.585 | 0.213 [-1.899, 0.431] |
| log(REM  respiratory disturbances) **→** | Long-delay | -1.262±0.629 | **0.049* [-2.517, -0.007**] |

p<0.05 significant predictor indicated by multiple regression model

**Table S30: Multiple regression models results of total number of oxyhemoglobin desaturations in REM sleep on RAVLT performance while controlling for *APOE4* status**

| **Predictor** | **Outcome** | **Unstandardized b ± SEM** | **Predictor p [95%CI]** |
| --- | --- | --- | --- |
| log(REM oxyhemoglobin desaturations) **→** | Total learning | -4.128±2.266 | 0.073 **[**-8.647**,** 0.391] |
| log(REM oxyhemoglobin desaturations) **→** | Short-delay | -0.941±0.649 | 0.151 [-2.234**,** 0.352] |
| log(REM oxyhemoglobin desaturations) **→** | Long-delay | -1.457±0.711 | **0.044* [**-2.875**,** -0.039**]** |

p<0.05 significant predictor indicated by multiple regression model

**Table S31: Multiple regression models results of total number of oxyhemoglobin desaturations in REM sleep on RAVLT performance while controlling for parental history of AD**

| **Predictor** | **Outcome** | **Unstandardized**  **b ± SEM** | **Predictor p [95%CI]** |
| --- | --- | --- | --- |
| log(REM oxyhemoglobin desaturations) **→** | Total learning | **-4.571±2.100** | **0.033 [-8.756, -0.385]** |
| log(REM oxyhemoglobin desaturations) **→** | Short-delay | -0.874±0.645 | 0.179 [-2.159, 0.411] |
| log(REM oxyhemoglobin desaturations) **→** | Long-delay | **-1.433±0.694** | **0.043* [-2.817, -0.048]** |

p<0.05 significant predictor indicated by multiple regression model

**Table S32. Multiple regression model results of OSA features *×* AD risk factors interactions (predictors) on RAVLT total learning (*APOE4* as a covariate in model)**

| **Predictor** | **Unstandardized b ± SEM** | **Predictor p [95%CI]** |
| --- | --- | --- |
| **log(REM AHI) *×* age** | -0.250±0.257 | 0.335 [-0.762, 0.263] |
| **log(REM AHI) *×* sex** | -1.771±3.757 | 0.639 [-9.264, 5.722] |
| **log(REM AHI) *×* APOE4** | -2.728±3.865 | 0.483 [-10.436, 4.981] |
|  |  |  |
| **log(REM RDI) *×* age** | -0.208±0.305 | 0.498 [-0.816, 0.401] |
| **log(REM RDI) *×* sex** | 2.258±4.253 | 0.597 [-6.224, 10.740] |
| **log(REM RDI) *×* APOE4** | -5.999±4.169 | 0.155 [-14.314, 2.316] |
|  |  |  |
| **log(REM ODI) *×* age** | -0.325±0.258 | 0.211 [-0.839, 0.189] |
| **log(REM ODI) *×* sex** | -3.694±4.307 | 0.394 [-12.285, 4.897] |
| **log(REM ODI) *×* APOE4** | -5.610±4.527 | 0.219 [-14.639, 3.419] |
|  |  |  |
| **log(REM AHI:NREM AHI) *×* age** | -0.233±0.427 | 0.587 [-1.086, 0.619] |
| **log(REM AHI:NREM AHI) *×* sex** | -7.632±5.043 | 0.135 [-17.689, 2.426] |
| **log(REM AHI:NREM AHI) *×* APOE4** | -3.252±5.924 | 0.585 [-15.066, 8.562] |
|  |  |  |
| **log(REM RDI:NREM RDI) *×* age** | -0.005±0.338 | 0.987 [-0.679, 0.668] |
| **log(REM RDI:NREM RDI) *×* sex** | -0.334±5.673 | 0.953 [-11.649, 10.982] |
| **log(REM RDI:NREM RDI) *×* APOE4** | -10.672±5.739 | 0.067 [-22.118, 0.774] |
|  |  |  |
| **log(REM ODI:NREM ODI) *×* age** | -0.536±0.406 | 0.190 [-1.345, 0.272] |
| **log(REM ODI:NREM ODI) *×* sex** | -3.063±6.053 | 0.614 [-15.136, 9.009] |
| **log(REM ODI:NREM ODI) *×* APOE4** | -14.054±6.277 | **0.028* [-26.573, -1.536]** |

p<0.05 significant predictor indicated by multiple regression model

**Table S33. Multiple regression model results of OSA features *×* AD risk factors interactions (predictors) on RAVLT total learning (parental history of AD as a covariate)**

| **Predictor** | **Unstandardized b ± SEM** | **Predictor p [95%CI]** |
| --- | --- | --- |
| **log(REM AHI) *×* age** | -0.355±0.239 | 0.141 [-0.831, 0.121] |
| **log(REM AHI) *×* sex** | -2.038±3.479 | 0.560 [-8.975, 4.899] |
| **log(REM AHI) *×* parental history of AD** | -1.677±3.447 | 0.628 [-8.549, 5.195] |
|  |  |  |
| **log(REM RDI) *×* age** | -0.395±0.284 | 0.168 [-0.962, 0.171] |
| **log(REM RDI) *×* sex** | 1.273±3.946 | 0.748 [-6.595, 9.141] |
| **log(REM RDI) *×* parental history of AD** | -4.198±3.733 | 0.265 [-11.641, 3.246] |
|  |  |  |
| **log(REM ODI) *×* age** | -0.473±0.233 | **0.046* [-0.938, -0.008]** |
| **log(REM ODI) *×* sex** | -3.608±3.962 | 0.365 [-11.508, 4.291] |
| **log(REM ODI) *×* parental history of AD** | -2.354±4.320 | 0.588 [-10.968, 6.261] |
|  |  |  |
| **log(REM AHI:NREM AHI) *×* age** | -0.246±0.402 | 0.543 [-1.047, 0.556] |
| **log(REM AHI:NREM AHI) *×* sex** | -6.564±4.501 | 0.149 [-15.539, 2.411] |
| **log(REM AHI:NREM AHI) *×* parental**  **history of AD** | -0.186±4.992 | 0.970 [-10.139, 9.768] |
|  |  |  |
| **log(REM RDI:NREM RDI) *×* age** | -0.115±0.318 | 0.719 [-0.749, 0.519] |
| **log(REM RDI:NREM RDI) *×* sex** | -1.942±5.149 | 0.707 [-12.208, 8.324] |
| **log(REM RDI:NREM RDI) *×* parental**  **history of AD** | -6.138±4.849 | 0.210 [-15.807, 3.531] |
|  |  |  |
| **log(REM ODI:NREM ODI) *×* age** | -0.617±0.376 | 0.105 [-1.367, 0.133] |
| **log(REM ODI:NREM ODI) *×* sex** | -3.165±5.475 | 0.565 [-14.083, 7.753] |
| **log(REM ODI:NREM ODI) *×* parental**  **history of AD** | -3.689±5.288 | 0.488 [-14.232, 6.855] |

p<0.05 significant predictor indicated by multiple regression model

**Table S34. Multiple regression model results of OSA features *×* AD risk factors interactions (predictors) on RAVLT long-delay recall (*APOE4* as a covariate in model)**

| **Predictor** | **Unstandardized b ± SEM** | **Predictor p [95%CI]** |
| --- | --- | --- |
| **log(REM AHI) *×* age** | -0.066±0.079 | 0.409 [-0.223 0.092] |
| **log(REM AHI) *×* sex** | -0.952±1.147 | 0.409 [-3.240, 1.336] |
| **log(REM AHI) *×* APOE4** | 0.284±1.188 | 0.812 [-2.085, 2.653] |
|  |  |  |
| **log(REM RDI) *×* age** | -0.073±0.096 | 0.448 [-0.265, 0.118] |
| **log(REM RDI) *×* sex** | -0.260±1.341 | 0.847 [-2.934, 2.413] |
| **log(REM RDI) *×* APOE4** | -0.452±1.330 | 0.735 [-3.105, 2.201] |
|  |  |  |
| **log(REM ODI) *×* age** | -0.093±0.082 | 0.258 [-0.256, 0.070] |
| **log(REM ODI) *×* sex** | -1.338±1.361 | 0.329 [-4.052, 1.376] |
| **log(REM ODI) *×* APOE4** | -0.459±1.447 | 0.752 [-3.345, 2.427] |
|  |  |  |
| **log(REM AHI:NREM AHI) *×* age** | -0.017±0.126 | 0.891 [-0.268, 0.234] |
| **log(REM AHI:NREM AHI) *×* sex** | -2.704±1.472 | 0.071 [-5.641, 0.232] |
| **log(REM AHI:NREM AHI) *×* APOE4** | -0.660±1.744 | 0.706 [-4.139, 2.819] |
|  |  |  |
| **log(REM RDI:NREM RDI) *×* age** | 0.038±0.104 | 0.717 [-0.169, 0.244] |
| **log(REM RDI:NREM RDI) *×* sex** | -1.094±1.737 | 0.531 [-4.559, 2.371] |
| **log(REM RDI:NREM RDI) *×* APOE4** | **-3.752±1.748** | **0.035* [-7.239, -0.265]** |
|  |  |  |
| **log(REM ODI:NREM ODI) *×* age** | -0.091±0.125 | 0.467 [-0.341, 0.158] |
| **log(REM ODI:NREM ODI) *×* sex** | -1.997±1.837 | 0.281 [-5.661, 1.667] |
| **log(REM ODI:NREM ODI) *×* APOE4** | -3.361±1.944 | 0.088 [-7.238, 0.515] |

p<0.05 significant predictor indicated by multiple regression model

**Table S35. Multiple regression model results of OSA features *×* AD risk factors interactions (predictors) on RAVLT long-delay recall (parental history of AD as a covariate in model)**

| **Predictor** | **Unstandardized b ± SEM** | **Predictor p [95%CI]** |
| --- | --- | --- |
| **log(REM AHI) *×* age** | -0.100±0.078 | 0.202 [-0.255, 0.055] |
| **log(REM AHI) *×* sex** | -1.073±1.124 | 0.343 [-3.315, 1.169] |
| **log(REM AHI) *×* parental history of AD** | -0.590±1.118 | 0.599 [-2.819, 1.639] |
|  |  |  |
| **log(REM RDI) *×* age** | -0.126±0.095 | 0.187 [-0.315, 0.063] |
| **log(REM RDI) *×* sex** | -0.641±1.310 | 0.626 [-3.254, 1.972] |
| **log(REM RDI) *×* parental history of AD** | -1.391±1.241 | 0.266 [-3.866, 1.083] |
|  |  |  |
| **log(REM ODI) *×* age** | -0.143±0.079 | 0.072 [-0.300, 0.013**]** |
| **log(REM ODI) *×* sex** | -1.332±1.328 | 0.319 [-3.980, 1.315] |
| **log(REM ODI) *×* parental history of AD** | -0.335±1.452 | 0.818 [-3.230, 2.561] |
|  |  |  |
| **log(REM AHI:NREM AHI) *×* age** | -0.034±0.125 | 0.789 [-0.284, 0.216] |
| **log(REM AHI:NREM AHI) *×* sex** | -2.447±1.391 | 0.083 [-5.221, 0.328] |
| **log(REM AHI:NREM AHI) *×* parental**  **history of AD** | -1.036±1.548 | 0.506 [-4.123, 2.052] |
|  |  |  |
| **log(REM RDI:NREM RDI) *×* age** | 0.003±0.103 | 0.978 [-0.202, 0.208] |
| **log(REM RDI:NREM RDI) *×* sex** | -1.523±1.654 | 0.360 [-4.820, 1.774] |
| **log(REM RDI:NREM RDI) *×* parental**  **history of AD** | -2.905±1.545 | 0.064 [-5.985, 0.175] |
|  |  |  |
| **log(REM ODI:NREM ODI) *×* age** | -0.126±0.122 | 0.306 [-0.369, 0.117] |
| **log(REM ODI:NREM ODI) *×* sex** | -1.619±1.749 | 0.358 [-5.106, 1.868] |
| **log(REM ODI:NREM ODI) *×* parental history of AD** | -0.970±1.697 | 0.569 [-4.353, 2.413] |

p<0.05 significant predictor indicated by multiple regression model

**Table S36.** **Contrasts comparing the slopes of the associations between**

**REM RDI and RAVLT total learning for the three AD risk factor groups**

| 0 risk factors = No parental history of AD or *APOE4*+  1 risk factor = Parental history of AD or *APOE4*+  2 risk factors = Parental history of AD and *APOE4*+ | | | |
| --- | --- | --- | --- |
| **Contrasts** | **Estimate** | **SE** | **p-value** |
| 0 risk factors – 1 risk factor | -1.8 | 4.31 | 0.91 |
| 0 risk factors – 2 risk factors | 10.7 | 5.20 | 0.11 |
| 0 risk factors – 1 risk factors | 12.5 | 4.38 | **0.02*** |
| *p<0.05 Tuckey’s corrected p-value | | | |

**Table S37. Contrasts comparing the slopes of the associations between**

**RDI ratio and RAVLT total learning for the three AD risk factor groups**

| 0 risk factors = No parental history of AD or *APOE4*+  1 risk factor = Parental history of AD or *APOE4*+  2 risk factors = Parental history of AD and *APOE4*+ | | | |
| --- | --- | --- | --- |
| **Contrasts** | **Estimate** | **SE** | **p-value** |
| 0 risk factors – 1 risk factor | 2.3 | 5.19 | 0.90 |
| 0 risk factors – 2 risk factors | 22.3 | 7.61 | **0.01*** |
| 0 risk factors – 1 risk factors | 20.0 | 6.79 | **0.01*** |
| *p<0.05 Tuckey’s corrected p-value | | | |

**Table S38.** **Contrasts comparing the slopes of the associations between**

**ODI ratio and RAVLT total learning for the three AD risk factor groups**

| 0 risk factors = No parental history of AD or APOE4+  1 risk factor = Parental history of AD or APOE4+  2 risk factors = Parental history of AD and APOE4+ | | | |
| --- | --- | --- | --- |
| **Contrasts** | **Estimate** | **SE** | **p-value** |
| 0 risk factors – 1 risk factor | -1.57 | 5.57 | 0.96 |
| 0 risk factors – 2 risk factors | 19.01 | 7.68 | **0.04*** |
| 0 risk factors – 1 risk factors | 20.58 | 6.90 | **0.01*** |
| *p<0.05 Tuckey’s corrected p-value | | | |

**Table S39. Contrasts comparing the slopes of the associations between**

**RDI ratio and RAVLT long-delay recall for the three AD risk factor groups**

| 0 risk factors = No parental history of AD or *APOE4*+  1 risk factor = Parental history of AD or *APOE4*+  2 risk factors = Parental history of AD and *APOE4*+ | | | |
| --- | --- | --- | --- |
| **Contrasts** | **Estimate** | **SE** | **p-value** |
| 0 risk factors – 1 risk factor | 1.29 | 1.65 | 0.71 |
| 0 risk factors – 2 risk factors | 7.75 | 2.42 | **0.01*** |
| 0 risk factors – 1 risk factors | 6.45 | 2.16 | **0.01*** |

*p<0.05 Tuckey’s corrected p-value
