# Supplementary material for: Older adults at greater risk for Alzheimer’s disease show stronger associations between sleep apnea severity in REM sleep and verbal memory
Source: Alzheimers Res Ther. 2024 May 9;16:102. doi: 10.1186/s13195-024-01446-3 (PMC11080222; doi:10.1186/s13195-024-01446-3)
Supplement: Supplementary file 2 — Supplementary Material 2. [file 13195_2024_1446_MOESM2_ESM.docx]

**SUPPLMENTARY FIGURES: Older adults at greater risk for Alzheimer’s disease show stronger associations between sleep apnea severity in REM sleep and verbal memory**

Kitty K. Lui^1,2^, Abhishek Dave^2,3^, Kate E. Sprecher^4-7^, Miranda G. Chappel-Farley^8,9^, Brady A. Riedner^10^, Margo B. Heston^6,7^, Chase E. Taylor^11^, Cynthia M. Carlsson^6,7,12,13^, Ozioma C. Okonkwo^6,7,12,13^, Sanjay Asthana^6,7,12,13^, Sterling C. Johnson^6,7,12,13^, Barbara B. Bendlin^6,7,12,13^, Bryce A. Mander^2,3,9*^, Ruth M. Benca^2,5,9,10,14*^

^1^San Diego State University/University of California San Diego, Joint Doctoral Program in Clinical Psychology, San Diego, CA, USA

^2^Department of Psychiatry and Human Behavior, University of California, Irvine, CA, USA

^3^Department of Cognitive Sciences, University of California, Irvine, CA, USA

^4^Department of Population Health Sciences, University of Wisconsin-Madison, Madison, WI, USA

^5^Neuroscience Training Program, University of Wisconsin-Madison, Madison, WI, USA

^6^Department of Medicine, University of Wisconsin-Madison, Madison, WI, USA

^7^Wisconsin Alzheimer's Disease Research Center, University of Wisconsin-Madison, Madison, WI, USA

^8^Department of Neurobiology and Behavior, University of California, Irvine, CA, USA

^9^Center for the Neurobiology of Learning and Memory, University of California, Irvine, CA, USA

^10^Department of Psychiatry, University of Wisconsin-Madison, Madison, WI, USA

^11^Department of Neuroscience, University of Kentucky, Lexington, KY, USA

^12^Wisconsin Alzheimer’s Institute, Madison, WI, USA

^13^Geriatric Research Education and Clinical Center, Wm. S. Middleton Veterans Hospital, Madison, WI, USA

^14^Department of Psychiatry and Behavioral Medicine, Wake Forest University, Winston-Salem, NC, USA

*Correspondence should be addressed to:

Ruth M. Benca, M.D., Ph.D., rbenca@wakehealth.edu | (336) 716-2911

Bryce A. Mander, Ph.D., [bmander@uci.edu](mailto:bmander@uci.edu) | (949) 824-6742


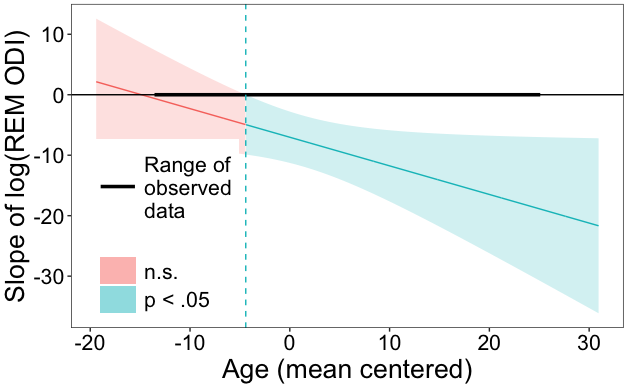


**Figure S1.** Johnson-Neyman plot demonstrating the range of the sample in which age significantly moderates the relationship between REM ODI and RAVLT total learning (in green). In 80% of the sample, more oxyhemoglobin desaturations during REM sleep were related to worse total learning.


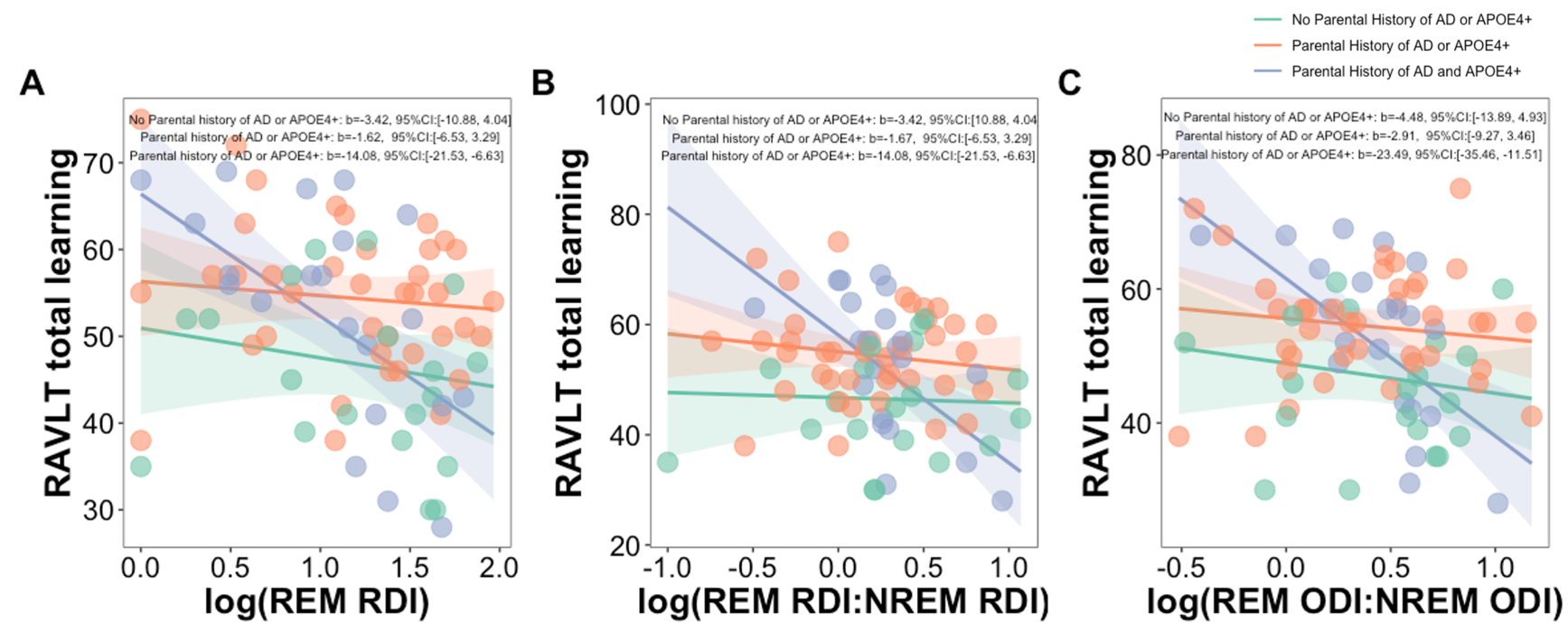


**Figure S2.** The moderating effects of AD risk factor groups on the associations between OSA events in REM sleep and verbal memory performance. AD risk factor groups were defined as 1) no parental history of AD or *APOE4* positive, 2) either parental history of AD or *APOE4* positive, and 3) have both parental history of AD and *APOE4* positive. (A) In only people with both parental history of AD and *APOE4* positive, higher REM RDI was significantly associated with lower RAVLT total learning scores. (B) In only people with both AD risk factors, higher REM-NREM RDI ratio was significantly associated with lower RAVLT total learning. (C) In only people with both AD risk factors, higher REM-NREM ODI ratio was significantly associated with lower RAVLT total learning.


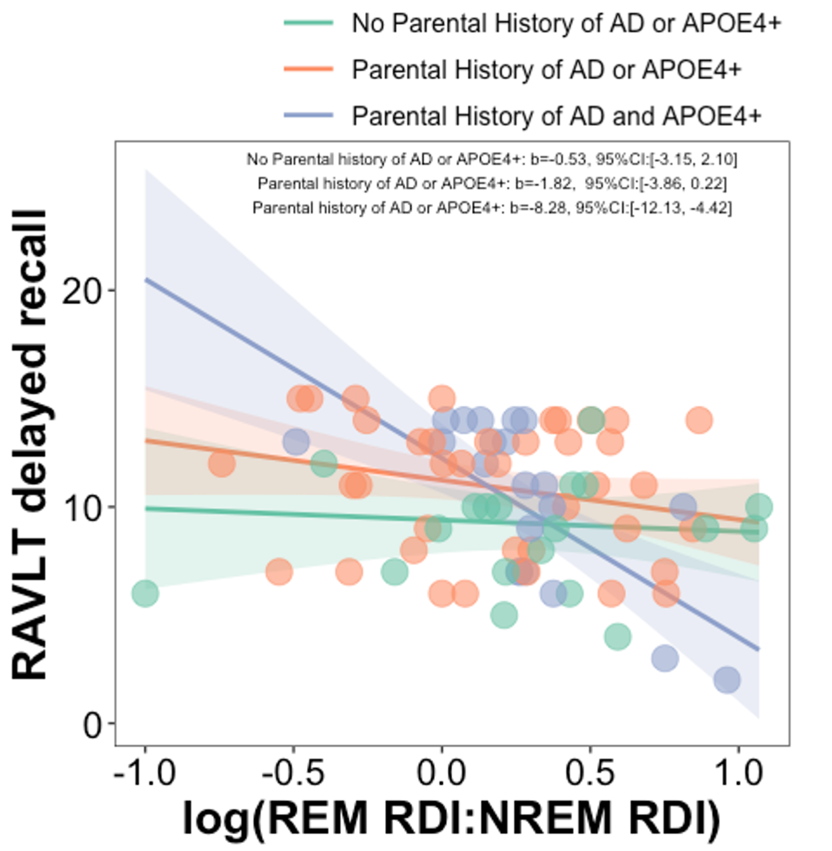


**Figure S3.** In only people with parental history of AD and *APOE4* positivity, higher REM-NREM RDI ratio was significantly associated with lower RAVLT delayed recall scores.
